# Development of the Manchester framework for the evaluation of emergency department pharmacy services

**DOI:** 10.1007/s11096-022-01403-w

**Published:** 2022-04-21

**Authors:** D Greenwood, MP Tully, S Martin, D Steinke

**Affiliations:** 1 Division of Pharmacy and Optometry, University of Manchester, Oxford Road, M13 9PT Manchester, UK; 2 School of Medicine, Anglia Ruskin University, Bishop Hall Lane, CM1 1SQ Chelmsford, UK; 3 School of Pharmacy and Medical Sciences, University of Bradford, Richmond Road, BD7 1DP Bradford, UK

**Keywords:** Advanced pharmacy practice, Emergency care, Outcomes research, Pharmacist practitioners, Quality evaluation

## Abstract

**Background:**

Many countries, including the United Kingdom, have established Emergency Department (ED) pharmacy services where some ED pharmacists now work as practitioners. They provide both traditional pharmaceutical care and novel practitioner care i.e. clinical examination, yet their impact on quality of care is unknown.

**Aim:**

To develop a framework of structures, processes and potential outcome indicators to support evaluation of the quality of ED pharmacy services in future studies.

**Method:**

Framework components (structures, processes and potential outcome indicators) were identified in three ways: from a narrative review of relevant international literature, and separate panel meetings with ED pharmacists and then other ED healthcare professionals. Structures and processes were collated into categories developed iteratively throughout data collection, with outcome indicators collated into six domains of quality as proposed by the Institute of Medicine. These raw data were then processed e.g. outcome indicators screened for clarity i.e. those which explicitly stated what would be measured were included in the framework.

**Results:**

A total of 190 structures, 533 processes, and 503 outcome indicators were identified. Through data processing a total of 153 outcome indicators were included in the final framework divided into the domains safe (32), effective (50), patient centred (18), timely (24), efficient (20) and equitable (9).

**Conclusion:**

The first framework specific to the quality evaluation ED pharmacy services, service evaluators should validate potential outcome indicators prior to their use. The minimum expected of a high-quality service should also be defined to enable interpretation of relevant measurements.

**Supplementary Information:**

The online version contains supplementary material available at 10.1007/s11096-022-01403-w.

## Impact statements

- The ‘Manchester Framework’ of outcome indicators can be used to support the quality evaluation of ED pharmacy services in the UK and internationally, with outcome measurements used to guide service developments.
- Additional outcome indicators are required to evaluate whether ED pharmacy services provide care that is patient centred, equitable and efficient, and thereby ensure all aspects of quality can be measured and quality concluded.

## Introduction

Pharmacists provide services to emergency departments (EDs) in many countries such as the United States [1], Saudi Arabia [2], Colombia [3] and the United Kingdom (UK) [4]. In the UK, in part due to a shortage of ED doctors and nurses, pharmacists have completed additional clinical training so to take on a greater role in patient care [5]. Termed ‘ED pharmacist practitioners’, they provide traditional pharmacy care e.g. medicines reconciliation [6], but also ‘practitioner’ care e.g. perform physical examinations and manage patients [5]. There has been some evaluation of ED pharmacy services, particularly in the United States [7, 8], but this is mostly limited to the more traditional activities of pharmacists in those settings. Overall, while at least 20 pharmacists now work as practitioners and provide care to patients in UK EDs [5], there has been no evaluation of the quality of those services. Evaluation of the quality of care provided by health services is important to identify areas requiring improvement, as well as ensure the appropriate use of resources [9, 10]. According to the Institute of Medicine [11], quality care is: safe, effective, patient-centred, timely, efficient and equitable.

Evaluation of the quality of care centres on the measurement of outcome indicators [12, 13]. Either qualitative or quantitative, indicator measurements are used to conclude the outcome of a service. It is also important to measure multiple outcome indicators together to ensure any observed change in measurement can be interpreted in the context of other measurements [14]. For example, if a desirable change is observed for one outcome indicator, with an undesirable change for another, these measurements can be considered together and the overall change deemed desirable or undesirable [15, 16]. The context of outcome measurements should also be considered to aid interpretation, specifically, the environment through which care is provided (structures) and actual care delivery (processes) [9].

In practice, quality evaluation first involves development of potential outcome indicators [17]. As outlined by Mainz and colleagues [14, 17], these should be clearly described, an ‘acceptable’ measurement defined (i.e. that expected of a high-quality service), and their reliability and validity tested. Reliability and validity testing are usually undertaken for a defined population, meaning the outcome indicator may only be reliable and valid for that population [16, 18].

Frameworks have been used to collate different indicators of structure, process and outcome and ensure a systems-based approach to quality evaluation [14, 19]. For example, in January 2019, the International Federation of Emergency Medicine (IFEM) published “An updated Framework on Quality and Safety in Emergency Medicine” [20]. The framework lists indicators of structure, process and outcome grouped into the six IoM domains of quality. While this and other frameworks exist to evaluate ED quality more generally, there is no framework specific to ED pharmacy services.

### Aim

To develop a framework of structures, processes and potential outcome indicators to support evaluation of the quality of ED pharmacy services in future studies.

### Ethics approval

No ethical approval was required as ED pharmacists and other healthcare professionals were asked non-sensitive questions that were within their professional competence unrelated to their employer. Panel members were aware of how their data would be used to create an evaluation framework.

## Method

Three data collection stages were chosen to identify potential outcome indicators, structures and processes, informed by methods used successfully by IFEM to develop their framework: a literature review (Stage 1), an expert panel with ED pharmacists (Stage 2) and finally an expert panel with other ED healthcare professionals (Stage 3). Campbell’s definitions of structure, process and outcome were used to guide all stages of data collection [11], with data collated into 12 separate tables (see electronic supplementary material A). Six of those tables were for outcome indicators, one for each IoM quality domain, with the indicators grouped into ‘topics of evaluation’ (i.e. outcomes). A sample of outcome indicators is presented later in Table 2.

### Literature review (Stage 1)

Five databases commonly used in Health Services Research were systematically searched for studies which had previously evaluated ED pharmacy services, with the objective of identifying and extracting potential outcome indicators, and structures and processes using the complete strategy (see electronic supplementary material B). Specific databases were: Cumulative Index of Nursing and Allied Health Literature’ (CINAHL); Web of Science Core Collection; MEDLINE; International Pharmaceutical Abstracts; and Embase. Based on the search objective and the Patient, Intervention, Comparison, Outcome (PICO) framework, three primary search terms were identified: emergency department; structure, process and outcome; and pharmacist. Preliminary searches with these terms were undertaken and the keywords of initial search results recorded and used as secondary search terms. Other secondary search terms were: synonyms of primary terms (e.g. structure, process, and outcome each included as individual terms); abbreviations; and international terminology. The completeness of the search strategy was tested by adding additional secondary search terms until the number of results stabilised.

After removal of duplicates, a total of 432 unique search results were identified from the databases. The reference lists of a systematic review [21] and narrative review [22] were examined to identify any further relevant literature. Titles and abstracts were then reviewed by the author for potential inclusion and 130 results thought potentially relevant were taken forward to full-text eligibility screening, including one study [4] identified from the reference lists of the reviews.

For full-text screening, the eligibility of studies was evaluated using the inclusion/exclusion criteria developed according to the study aim. Studies were reviewed and re-reviewed to ensure decisions made to include/exclude them were accurate. Of the 130 studies reviewed, 34 met the inclusion criteria and were taken forward to data extraction [4, 6, 23–54].

Eligibility was not checked by a second researcher. The quality of the studies as a whole was not checked as poor-quality studies could still have included high quality outcome indicators which would add to the framework.

#### Inclusion criteria

Studies included were those that:

- Measured the direct impact of pharmacists’ on the quality of care (i.e. direct from pharmacist to patient); or the indirect impact of pharmacists’ on the quality of care (i.e. outcomes via another healthcare professional); or the direct impact of a pharmacist on the activities of other healthcare professionals (i.e. a surrogate outcome).
- Measured ED pharmacist processes (i.e. no outcome indicators) and were UK studies.
- Measured outcomes which manifested and/or were measured within or outside the ED.
- Involved pharmacists who work in the ED, or units closely affiliated/named differently e.g. ED short stay unit.
- Involved the care of a general adult population (no less than 16 years of age), or if paediatric data were included then adult data were available for extraction separately..

As most studies of ED pharmacist impact are international, UK studies which only concerned ED pharmacist processes (i.e. no outcome indicators) were included to ensure the framework included UK relevant processes. While increasing the framework’s relevance to UK practice, this approach did not detract anything from its use in other countries.

#### Exclusion criteria

Studies excluded were those that:

- Suggested, rather than measured, the impact of ED pharmacists i.e. suggestions based on opinion or the extrapolation of previous research.
- Investigated a specialist ED e.g. for elderly or paediatric patients only.
- Had a ‘research pharmacist’ (i.e. a pharmacist who did not routinely provide patient care in the ED) carry out patient care processes in the ED e.g. for an intervention study..

**Fig. 1:**
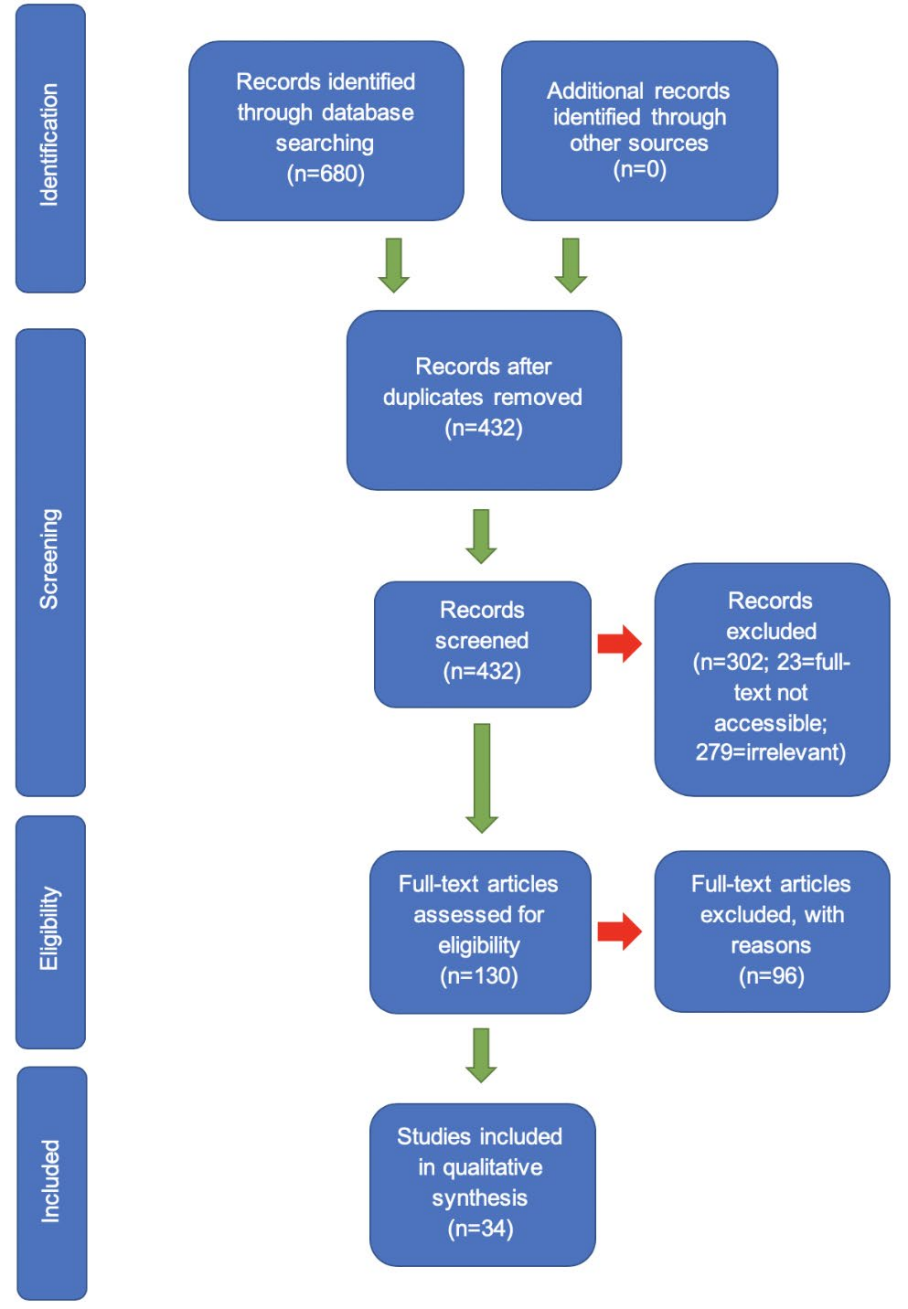
Summary of the stages of literature identification, screening, eligibility assessment and inclusion

For each study, structures, processes and outcome indicators were extracted and collated into the 12 data tables. Extraction was done independently by author DG but under the supervision of the other authors i.e. decisions made by DG were justified and discussed during meetings of all authors. As per the review inclusion criteria, outcome indicators of ED pharmacist’s indirect impact on patient care (i.e. via another healthcare professional) were included. Albeit indirect, these outcome indicators still suggested a relationship between pharmacist activity and outcomes. They were, however, collated separately for if it was later decided they should be excluded from the framework. An overall summary of literature processing is given in Fig. 1.

### ED pharmacist panel (Stage 2)

A panel of seven ED pharmacists was convened in October 2017 to identify further potential topics of evaluation (outcomes), outcome indicators, and processes. Many recurring structures had already been identified from the literature so were not sought in what would already be a busy meeting. Data sources which could be used to measure outcome indicators were also sought. Audio recorded and lasting two hours, the meeting was held at the University of Manchester (UoM) and hosted by the primary author with the support of authors DS and SM. The five pharmacists who participated face-to-face were all students of the UoM ‘Advanced Specialist Training in Emergency Medicine’ (ASTEM) programme which upskills ED pharmacists to provide practitioner care. Of those five pharmacists, four worked in EDs in the North West of England while one worked in Scotland. Two further ED pharmacists joined via Skype® (both audio and video), neither of whom were ASTEM students or graduates but did provide practitioner care. All participants had at least two years of experience working as a pharmacist and a postgraduate diploma in clinical pharmacy.

Using a data collection form, participants were first asked to record potential topics for evaluation (outcomes) of ED pharmacist services (both ‘traditional’ and ‘practitioner’ activities, as per a previously developed work typology) [55]. In turn, participants then contributed their ideas to the panel, which were collated on a flipchart visible to participants. Although asked to prioritise ideas not already on the flipchart, they could comment on existing suggestions. Next, and in small groups, participants were asked to consider the potential outcomes for two of the six IoM domains and suggest related processes. Processes were not fed back to the whole group or discussed, due to time constraints. After the meeting, each participant was forwarded all outcomes and related processes and asked to record potential indicators for each of the outcomes. Data sources that could be used to support evaluation of outcome indicators were also sought.

### Multidisciplinary panel (Stage 3)

A panel of five ED healthcare professionals was convened in March 2018 to identify further potential outcome indicators. Participants were two staff nurses; a nurse practitioner; a physiotherapist; and an occupational therapist. Physicians were invited but none were recruited. They were chosen due to their varied roles which could support identification of different indicators, and were recruited through professional networks or Twitter® and worked in UK EDs. Lasting two hours and facilitated by author DG, the meeting platform GoToMeeting® was used whereby data collection was presented interactively on screen.

First, outcomes identified from the literature and ED pharmacist panel were listed on screen for one of the IoM quality domains. Taking each outcome in turn, participants were asked to record potential outcome indicators with an example given for the first outcome listed to aid understanding of the task. Having recorded all their ideas (unlimited time permitted), participants were in turn asked to share their indicators with the group with these recorded on screen by DG. The process was then repeated for each outcome for the first IoM domain, and then for a further three domains. Due to time constraints, participants were forwarded outcomes of the final two IoM domains for consideration after the meeting. They were asked to return their suggestions to the meeting facilitator when convenient. Data sources which could be used to support evaluation of outcome indicators were also sought.

### Processing data and creation of the Manchester framework

Raw data were placed in tables of direct and indirect outcome indicators and any duplicates e.g. exactly the same outcome indicator identified from multiple data sources, combined. Then, all outcome indicators were screened with those which were vague i.e. more closely resembled a theme and did not explicitly state what would be measured, removed. To increase usability of the resultant framework, the more specific outcome indicators were collated into broader outcome indicators. For example, indicators for the safety domain ‘number of prescribing errors’ and ‘number of errors that involve high-risk medicine’ were collated under the broader outcome indicator ‘number of medication errors’, with those more specific indicators given as examples. Some outcome indicators were also re-worded to ensure clarity but also international relevance. For example, references made to ‘Trust’ (a term used to describe a collection of hospitals or health services in the UK) were reworded to ‘hospital’. For structures and processes, some examples of different categories were included in the final framework as examples which the user may wish to consider for evaluation studies. To that end, instructions for how to use the framework are also included, with reference made to a study which has previously evaluated the quality impact of an ED pharmacy service.

## Results

From the three stages of data collection and prior to data processing, a total of 399 direct outcome indicators, 103 indirect outcome indicators, 533 processes and 190 structures were identified (unique items), divided into 12 different categories (Table 1).

**Table 1:** The 12 raw data tables with the total number of unique items identified for each category, with examples

Data table	Component	Categories	No. items	Example
1	Structures	Emergency department	176	Number of visits
2		Organisation	14	Number of beds
3	Processes	Patient specific	470	Obtain medical history
4		General	63	Educate and train
5	Direct outcome indicators	Safe care	75	Pharmacist prescribing safety
6		Effective care	147	Value of an antimicrobial stewardship service
7		Patient centred care	30	Patient preference of therapy
8		Timely care	72	Review of ‘Sepsis Criteria’
9		Efficient care	42	Use of ‘Patients Own Drugs’ (PODs)
10		Equitable care	33	Equal treatment based on condition
11	Indirect outcome indicators	Acceptance/rejection rate given	84	Doctor acceptance of pharmacist intervention related to a prescribed overdose
12		Acceptance/rejection rate not given	19	Reduced costs as a result of adverse drug event prevention

### The framework

The ‘Manchester framework for the evaluation of ED pharmacy services’ (see electronic supplementary material C) comprises three sections. In Section A, of the 502 unique potential outcome indicators identified (399 direct, 103 indirect), 322 had clarity (i.e. explicitly stated what would be measured) and were collated to give the final total of 153. The 153 potential outcome indicators are presented for the six quality domains: safety (32 potential outcome indicators), effectiveness (50), patient centred (18), timely (24), efficient (20), and equitable (9). Table 2 presents example potential outcome indicators for each of the domains, grouped into specific areas of evaluation.

**Table 2:** Example indicators to evaluate ED pharmacy services for each domain i.e. when a pharmacist is part of the care team

Domain	To evaluate…	You could measure…
Safety	The safety/safety impact of medication history taking/medicines reconciliation by a pharmacist	• Whether medicines reconciliation was undertaken • Number of errors in medication history/reconciliation taken by pharmacists e.g. allergy status not documented • How medicine(s) administered in the ED differ from the medicine(s) recorded in the medication history/reconciliation • How medicines reconciliation performed differs from an optimal reconciliation
	Pharmacist prescribing	• Number of prescribing errors • Number of adverse events for pharmacist prescriptions • Number of interventions made for pharmacist prescriptions • Re-attendance to ED or General Practitioner and reasons for re-attendance
Effectiveness	Effective use of medicine(s) when a pharmacist is part of the care team e.g. prescribes or reviews treatment	*This will depend on the type of medicine being evaluated. For example*: *To evaluate the use of Tissue Plasminogen Activator, you could measure relevant clinical indicators such as*: • Whether patient blood pressure is reduced to the required level *To evaluate the use of analgesia e.g. for trauma or post-intubation, you could measure relevant clinical indicators such as*: • Number of patients who receive analgesia • Change, and rate of change, in patient pain scores
	Medication interventions by pharmacists	• Whether doctors/nurses acknowledge pharmacist interventions • Whether doctors accept pharmacists’ medication interventions e.g. suggestions to change a medicine, dose, or time of administration, or prescribe an omitted medicine • Whether nurses accept pharmacists’ medication interventions e.g. to change the administration rate of an intra-venous medicine, or the method used to prepare a medicine
Patient centred	Whether the formulation of prescribed medicine is appropriate	• Whether patients are able to take the prescribed medicine • Patient opinion of formulation administered, including the opinions of particular groups e.g. children or those with a nasogastric tube • Change in patient compliance if a cheaper medicine (e.g. generic) is prescribed • Whether alternative formulations are sourced for patients
Timeliness	Time to time-critical medicines e.g. antimicrobials, analgesia, Tissue Plasminogen Activator and anti-Parkinsonians	*Relevant clinical indicators* • Time between arrival at ED or bed (bay) allocation to stages of medicine use e.g. from arrival to antimicrobial prescription or administration • Time between stages of medicine use e.g. time between administration of analgesia by ambulance service and again in the ED; time between antimicrobial prescription and administration
	Length of ED and hospital stay	• Length of ED stay e.g. overall and in different areas such as ‘majors’ or ‘resus’ • Length of hospital stay both overall and for different inpatient departments • Length of hospital stay for specific clinical groups e.g. those who experience medicines related admissions
Efficiency	Use of ‘Patient’s Own Drugs’ (PODs) to reduce use of hospital medicines	• Number of patients who have/use PODs in the ED/inpatient wards if admitted to hospital • Expenditure on PODs (i.e. regular medicines) compared with acute medication (i.e. newly prescribed in ED) • Whether patient’s prescriptions in primary care are regular (i.e. routine supply by community pharmacy)
Equitability	The equitability of clinical governance	• Pharmacist contribution to ED clinical governance meetings and investigations e.g. of incidents • Pharmacist contribution to development and review of guidelines and protocols e.g. how many they helped to develop

Section B of the framework lists 11 example structures and 16 example processes. Some of these are presented below in Table 3.

**Table 3:** Structures of the ED* and patient specific processes** - for the evaluation of ED pharmacist impact you could consider…

	Category	Example
Structures of the ED	Type of department and areas within	Type 1 ED (UK NHS nomenclature for a major ED) with resuscitation facilities
	Size of department	Number of visits
	Specialisms of department	Trauma centre
	Facilities	Care pathways
	Pharmacy facilities	Pre-packed medicines
	Recommended resources	Medicines formulary
Patient specific processes	History taking	Took drug histories Took a full medical history
	Clinical examinations	Performed clinical examinations Reviewed the findings of clinical examinations
	Investigations, tests and procedures	Reviewed the results of urine cultures Reviewed the results of pregnancy tests
	Diagnosis	Diagnosed patients Educated and trained patients about their diagnosis

*Some structures of the wider care organisation were also identified and so should also be considered e.g. the type of hospitals

**General processes were also identified and should also be considered e.g. guideline development

In Section C, an example study [4] which measured outcome indicators and considered structures and processes is given to demonstrate how components of the framework could be used. Some examples of data sources are also given, such as: medical notes, medication administration records, pain and function score charts, and ‘near-miss’ error logs.

## Discussion

### Statement of key findings

Although there are already frameworks which can be used to evaluate the quality of ED care more generally, this study is the first specific to ED pharmacy services. The inclusion of international literature, and use of the IoM quality domains [11] to guide identification of structures, processes and potential outcome indicators, means the framework has global relevance. At least some items e.g. particular outcome indicators, should be relevant to the pharmacy services of any given country. With respect to the UK where some ED pharmacists now work as practitioners, the framework can be used to evaluate both traditional and new ‘practitioner’ care.

Supporting a system-based approach to evaluation, the framework can be used to evaluate different indicators together by pharmacists with researchers. Potential outcome indicators are grouped into the different domains of quality should more focused evaluation be desired e.g. to purposely measure only the safety of care. Further, through narrative and use of an example study, the framework explains how to evaluate quality including how processes can be used to identify outcome indicators sensitive to said processes. As has been stressed by other researchers [9, 13], the importance of processes and structures in contextualising outcome measurements is also described with examples of those components given.

Fewer potential outcome indicators were identified for the ‘equitable’, ‘efficient’ and ‘patient-centred’ domains, than for the other three. For equitable and efficient, no indicators were identified from the literature. The IoM recognise that the equitable and efficient domains, and to a lesser extent patient-centred, are often neglected by evaluators [56]. Indeed, some organisations have opted not to include the equitable and efficient domains in their definition of quality e.g. the Organisation for Economic Co-operation and Development [57]. With regards to ED pharmacy services, these three domains might be more challenging to consider or investigate than the others, or a lower priority for evaluation. Given that the IoM domains are themselves supposed to be ‘equitable’ i.e. six aims for healthcare improvement [56], further identification of indicators for those neglected domains is warranted. However, it could be that for ED pharmacy services, there are fewer potential outcome indicators to be identified for those quality domains typically neglected.

Considering the methods used to develop the framework, many of the potential outcome indicators suggested by the expert panels more closely resembled themes i.e. no detail as to what might be measured. While expert contribution was valuable, particularly to gain more current data e.g. outcome indicators for novel practitioner care, panel meetings took longer than anticipated, perhaps due to the complex nature of quality. Further, many outcome indicators suggested lacked clarity and instead more closely resembled themes. With respect to indicators of ‘indirect outcomes’ i.e. outcomes via another healthcare professional, these were included as no reason to exclude them from the framework was identified throughout development. Much of pharmacists’ work is to influence other healthcare professionals e.g. prescribing error interventions, with a change in outcome still possible albeit indirectly.

### Strengths and weaknesses

A strength of this study is the use of different data collection methods to identify varied framework components, enabling a large range of future evaluation studies. Another strength, literature databases were searched systematically, meaning most – if not all – relevant studies from five databases were included. There are also several limitations to this study. First, experts were all UK healthcare professionals which may mean the framework is less relevant to other countries. Further, five of the seven pharmacists who participated were ASTEM students who practiced in the North West of England, potentially meaning the framework is more tailored to practices in that region. The inclusion of literature from different countries countered these limitations, increasing the international relevance of the framework. Another limitation, due to meetings taking longer than planned, final tasks were often completed independently by participants after the meeting. This may have limited the amount and quality of data collected as there was no opportunity for group discussion of those final tasks.

A third limitation, all four authors are pharmacists and pharmacy practice researchers whom could be biased in favour of pharmacists. For example, particular studies could have been favoured and included in the final framework with others discarded. To ensure a consistent approach, explicit inclusion and exclusion criteria were developed to screen studies. The potential for bias should also be an important consideration for those who use the framework and evaluate ED pharmacy services. Evaluation teams should be multidisciplinary to ensure different perspectives are considered [17].

### Interpretation

The Manchester framework provides a starting point to evaluate ED pharmacy services. Although comprised of potential outcome indicators which require validation prior to their use, their eventual measurement could indicate the quality of ED pharmacy services. If outcome measurements are undesirable, services could be modified e.g. through development of specific, targeted interventions. Re-measurement can then be used to conclude whether changes have had a positive or negative impact on outcomes. Having been developed from international literature, if used by service providers in different countries, the framework provides global alignment for evaluation.

### Further research

Further research should seek to develop additional outcome indicators which could be used to measure the extent to which ED pharmacy services are equitable, efficient and patient-centred domains. These domains are currently under-represented in the framework – something which is by no means limited to evaluation of ED pharmacy services, but rather, is an issue of quality evaluation more broadly. Given that expert panels often contributed themes rather than explicit outcome measures, a recommendation for others seeking to identify explicit outcome indicators would be to use longer or several sequential meetings could prove more effective.

Future research should use potential indicators to evaluate the quality of ED pharmacy services. For example, to evaluate safety, evaluators should first choose one or more relevant outcome indicators and validate them. Then, an ‘acceptable’ measurement for each chosen indicator should be defined i.e. the minimum acceptable standard of service. Progressing to actual measurement, given the complexities of systems-based outcome measurement, evaluation teams should be multidisciplinary and ideally involve researchers with relevant expertise. Finally, dissemination of measurements and resultant service modifications will increase our understanding of what constitutes a high-quality ED pharmacy service.

## Conclusion

Building on the experiences and methods of more general ED quality evaluation frameworks, the Manchester framework is the first exclusively concerned with the evaluation of ED pharmacy services. Prior to their use, outcome indicators should be treated as *potential outcome indicators* which first require further development i.e. validation, with standards to interpret measurements also defined. When evaluating ED pharmacy services, all quality domains should be assessed and not just those that are straightforward to measure, at the expense of those that are neglected. After all, quality health services are not only safe, effective and timely, but also patient centred, efficient and equitable.

## Electronic Supplementary Material

Below is the link to the electronic supplementary material.

Supplementary Material 1

Supplementary Material 2

Supplementary Material 3
