# Supplementary material for: Development of the Manchester framework for the evaluation of emergency department pharmacy services
Source: Int J Clin Pharm. 2022 Apr 21;44(4):930–8. doi: 10.1007/s11096-022-01403-w (PMC9393142; doi:10.1007/s11096-022-01403-w)
Supplement: Supplementary file 1 — Supplementary Material 1 [file 11096_2022_1403_MOESM1_ESM.docx]

## Appendix A. Data tables

The structure of the 12 separate data tables is presented in Figure 1 to aid navigation.

Figure 1. structure of the 12 separate data tables

Figure 1: structure of the Quality Evaluation Framework

Figure 1. structure of the Quality Evaluation Framework

Figure 1: structure of the Quality Evaluation Framework

**Table 6. Direct outcomes and outcome indicators: Effective care**

**Table 6. Direct outcomes and outcome indicators: Effective care**

**Table 7. Direct outcomes and outcome indicators: Patient centred care**

**Table 7. Direct outcomes and outcome indicators: Patient centred care**

**Table 8. Direct outcomes and outcome indicators: Timely care**

**Table 8. Direct outcomes and outcome indicators: Timely care**

**Table 9. Direct outcomes and outcome indicators: Efficient care**

**Table 9. Direct outcomes and outcome indicators: Efficient care**

**Table 10. Direct outcomes and outcome indicators: Equitable care**

**Table 10. Direct outcomes and outcome indicators: Equitable care**

**Table 5. Direct outcomes and outcome indicators: Safe care**

**Table 5. Direct outcomes and outcome indicators: Safe care**

**Table 4. Processes: General**

**Table 4. Processes: General**

**Table 3. Processes: Patient specific**

**Table 3. Processes: Patient specific**

**Table 1. Structures: Emergency department**

**Table 1. Structures: Emergency department**

**Table 2. Structures: Organisation**

**Table 2. Structures: Organisation**

**Table 11. Indirect outcomes: *evaluated* influence on others**

**Table 11. Indirect outcomes: *evaluated* influence on others**

**Table 12. Indirect outcomes: *presumed* outcome due to influence on others**

**Table 12. Indirect outcomes: *presumed* outcome due to influence on others**

**Structures**

**Structures**

**Structures**

**Structures**

**Processes**

**Processes**

**Processes**

**Processes**

**Direct outcomes and indicators**

**Direct outcomes and indicators**

**Direct outcomes and indicators**

**Direct outcomes and indicators**

**Indirect outcomes**

**Indirect outcomes**

**Indirect outcomes**

**Indirect outcomes**

Table 1. Structures: Emergency Department

| **Level 1 structure** | **Level 2 structure** | **Level 3 structure** |
| --- | --- | --- |
| 1. **Type of department and areas within** | | |
| Type 1 | Majors (1-3) |  |
|  | Accompanying unit (4) |  |
|  | Resuscitation area (2) |  |
|  | Critical care (3) |  |
|  | Trauma area (3) |  |
|  | Observation unit (3) |  |
| Type 3 | Walk-in centre (1) |  |
|  | Minor injuries unit (1) |  |
|  | Fast track area (3, 5) |  |
|  | Urgent care (3) |  |
| 1. **Size of department** | | |
| Number of visits (1-3, 6-18) | Trauma patients (19) |  |
|  | Emergency admissions (18) |  |
| Number of beds (2, 3, 7, 10, 13-15, 20) |  |  |
| Large (21) |  |  |
| 1. **Specialisms of department** | | |
| Academic (8, 20, 21) |  |  |
| Burns (20) |  |  |
| Trauma (2, 9, 20) |  |  |
| Education for pharmacists (20) |  |  |
| Tertiary care (11, 21) |  |  |
| 1. **Facilities** | | |
| Beds | Dedicated acute in-patient beds in short-stay area (1) |  |
| Pharmacy | Satellite pharmacy (10) |  |
|  | Medicine’s storage (5) |  |
|  | Access to outpatient pharmacy (1) |  |
|  | Access to main hospital dispensary (1) |  |
|  | Pre-packed medicines (1) |  |
| 1. **Resources that should be used (EPS1)** | | |
| Formulary | Restricted formulary (EPEFFECT7) |  |
| Policies related to (EPEFFECT9): | Cost effective prescribing (EPEFFECT7) | Evidence why didn’t follow policy if applicable (EPEFFECT9) |
|  | Safeguarding (EPPC33) |  |
| Guidelines related to (EPEQ20): | Prescribing (EPEQ20) |  |
|  | Patient care (EPEQ20) |  |
|  | Integrated care (EPEFFECT29) |  |
|  | Critical care medicines (EPEFFECT29) |  |
| Pathways | Ambulatory care (EPT35) |  |
| Checklists (EPEFFIC37) |  |  |
| In-date sources (EPS3) |  |  |
| 1. **Patient population** | | |
| Acuity | Number of patients admitted to hospital (7, 9) (22) |  |
|  | Number of patients initially seen in resuscitation (10) |  |
|  | Number of patients initially seen in Majors (10) |  |
|  | Number of patients discharged home (22) |  |
|  | Average length of stay (22) |  |
| Condition | Medical patients (22) | Emergencies (2, 5) |
|  | Surgical patients (22) |  |
|  | Trauma patients (2) | Number of trauma patients |
| 1. **Care delivery** | | |
| Ward rounds (1, 4) |  |  |
| 1. **Pharmacy systems for:** | | |
| Interventions | Electronic system (4) |  |
|  | Standardised forms (23) |  |
| If pharmacist unavailable | Over-ride requirement for pharmacist review of discharge prescriptions (23) |  |
| Triage to pharmacist | Electronic notification system | Patients who potentially have severe sepsis and septic shock (6) |
|  |  | Patients who have not been vaccinated for influenza this season (5) |
|  |  | Patients who are to be admitted to inpatient ward (16) |
|  | Medical and nursing staff |  |
| Prescribing | Electronic prescribing (2, 3) (EPS23) |  |
|  | Decision support alerts | Drug-allergies (3) |
|  |  | Drug-drug interactions (3) |
|  |  | Renal dosing (3) |
|  |  | Duplicate orders (3) |
|  | Antibiograms to support prescribing decisions (24) |  |
| Health records | Electronic health records (15) |  |
| Medicines reconciliation | Standardised tool (9, 25) |  |
| Drug history taking | Standardised tool (25) |  |
| 1. **Pharmacist service** | | |
| Location from where service provided | The ED (1-5, 7, 9, 12, 14, 16, 26, 27) |  |
|  | The wider hospital (1) |  |
|  | Central pharmacy (9) |  |
|  | Offsite (23) |  |
| Availability | Regular visits or dedicated full-time position (1-5, 7, 9, 12, 14, 16, 26, 27) (EPS23) |  |
|  | Out-of-hours (1) (EPS23) |  |
|  | Residency program (3) |  |
| History | Length of time service established (3) |  |
|  | Extensive knowledge of resources including medicines information sources (EPS1) |  |
| Pharmacist education: | Prescribing qualification (EPS23) |  |
| Pharmacist knowledge and skills related to: | Treatment | Guidelines (EPS24) |
|  | Medicines (EPS2) (EPS3) | When to give (EPS2) |
|  |  | Formulations (EPS2) (EPEFFECT8) |
|  |  | Specialist medicines (EPS23) |
|  |  | Can cause secondary illness (EPS4) |
|  |  | Number Needed to Treat (EPS4) |
|  |  | Number Needed to Harm (EPS4) |
|  | Communication | With central pharmacy department (EPS2) |
|  | Consultation skills (EPPC13) | With patient to understand how they are feeling (EPPC31) |
|  |  | With patient so they are at the centre of the consultation (EPPC31) |
|  | Processes | Pharmacy processes (EPS2) |
|  |  | Pain pathway (EPEFFECT10) |
|  |  | Locations of Standard Operating Procedures and pathways (EPS24) |
|  |  | Ambulatory care pathways (EPT35) |
| Capacity | Time available to provide medicines reconciliation (EPS3) |  |
| Self-management | Organise time efficiently (EPEFFIC39) |  |
|  | Less time wasted (EPEFFIC41) |  |
| Pharmacy student / intern support (2, 5) |  |  |
| 1. **Other staff and their interaction with pharmacist** | | |
| Doctor (3-5, 10, 11, 20) | Helped EDPP through: | Clarification of information (9) |
|  | Collaborated with EDPP to: | Modify treatment plan (11) |
|  |  | Undertake medication therapy management and reconciliation (12) |
|  |  | Discussion of required medication adjustments (12) |
|  |  | Discussion of medication errors (12) |
|  | Helped by EDPP to: | Order tissue plasminogen activator (28) |
| Triage nurse | Helped EDPP through: | Indication of patients who require influenza vaccination (5) |
| Emergency nurse practitioners (1) |  |  |
| Nurse (3, 10, 20) |  |  |
| Doctor and nurse | Collaborated with EDPP to: | Review cultures and prescribed discharge antimicrobials (29) |
| General practitioners (1) | Helped EDPP through: | Confirmation of medication history (EPS3) |
|  |  | Confirmation of medication history for purposes of medicines reconciliation (EPS3) |
| Community pharmacist | Helped EDPP through: | Clarification of information (9) |
|  |  | Confirmation of whether patient adherent (12) |
|  |  | Supply of new antimicrobial prescription (7) |
| Emergency Medical Services | Prior to EDPP care: | Administered pain medication to patient prior to arrival (19) |
| 1. **Patient’s family or caregiver and their interaction with pharmacist** | | |
| Helped EDPP through: | Clarification of information (9) |  |
|  | Confirmation of whether patient adherent (12) |  |
|  | Confirmation of medication history (EPS3) |  |
|  | Confirmation of medication history for purposes of medicines reconciliation (EPS3) |  |
| 1. **Patient and their interaction with pharmacist** | | |
| Helped EDPP through: | Confirmation of whether adherent (12) |  |
|  | Confirmation of medication history (EPS3) |  |
|  | Confirmation of medication history for purposes of medicines reconciliation (EPS3) |  |
| Asked by EDPP to: | Bring home medicines in with them (EPS2) |  |

Table 2. Structures: Organisation

| **Level 1 Structure** | **Level 2 Structure** | **Level 3 Structure** |
| --- | --- | --- |
| Number of hospitals in Trust (7) |  |  |
| Type of hospital | Academic (6, 17) |  |
|  | Teaching (9, 25, 30) |  |
|  | Tertiary centre (6, 10) |  |
|  | Trauma centre (10) | Level 2 (12) |
|  | Specialty care units (10) | Level 3 (13) |
|  | Community (15, 19) |  |
|  | City hospital (16) |  |
|  | District general (16, 18) |  |
| Number of beds (9, 10, 12-14, 25) |  |  |
| Number of patients cared for per annum (2, 3, 5-18, 20, 22, 23, 27, 29) |  |  |

Table 3. Processes: Patient Specific

| **Level 1 Process** | **Level 2 Process** | **Level 3 Process** | **Level 4 Process** | **Level 5 process** |
| --- | --- | --- | --- | --- |
| **1. History** | | | | |
| Obtain | | | | |
| Medical history (5) | Height (9) |  |  |  |
|  | Weight (9) |  |  |  |
| Drug history (1, 25, 26, 31) | Questions | Use of prescription medicines (9) |  |  |
|  |  | Use of non-prescription products (9) |  |  |
|  |  | Use of herbal products (9) |  |  |
|  |  | Allergy status (9) | Confirmed timing (9) |  |
|  |  |  | Confirmed type of reaction (9) |  |
|  |  |  | Confirmed severity (9) |  |
|  | Technique | Consider patient specific issues to support accurate medication history (EPS3) |  |  |
|  |  | Extended questioning | About over the counter medicines (EPS3) |  |
|  |  |  | About herbal medicines (EPS3) |  |
|  |  | Use of open questions | Covering all types of medication (EPS3) |  |
|  |  | Use of closed questions | Covering all types of medication (EPS3) |  |
|  | Information sources | Use patients own drugs (EPS3) |  |  |
| Medicines reconciliation (9, 18, 25, 31) (EPS3) | Identification of discrepancies (9) |  |  |  |
|  | Check doses (EPEFFECT10) |  |  |  |
|  | Record social history | Identification of need for a compliance aid (16) |  |  |
|  | Intervention | Correction of discrepancies (9) |  |  |
|  |  | Identified medicines reconciliation had not been done and informed doctor (22) |  |  |
|  |  | Ask prescriber to prescribe missing medicines (EPS2) |  |  |
|  | Information sources | Use patients own drugs (EPS3) |  |  |
|  | Technique | Consideration of patient specific issues (EPS3) |  |  |
|  |  | Extended questioning | About over the counter medicines (EPS3) |  |
|  |  |  | About herbal medicines (EPS3) |  |
|  |  | Use of open questions | Covering all types of medication (EPS3) |  |
|  |  | Use of closed questions | Covering all types of medication (EPS3) |  |
| Review (22) | | | | |
| History of presenting complaint (7, 11) |  |  |  |  |
| Presenting complaint (12) |  |  |  |  |
| Medical history (7, 12) | Vaccination status | Tetanus (EPEFFECT26) |  |  |
| Drug history (12) | To check: | Whether error identified previously has been corrected (9) |  |  |
|  |  | Patient allergy status (11) |  |  |
|  |  | Patient’s adherence to medication (12) |  |  |
| Identify | | | | |
| From drug history: | Issues with regular medicines or recent acute medicines |  |  |  |
|  | Medication related admissions (EPT34) |  |  |  |
|  | Discrepancies | Noncompliance and medications not prescribed from drug history (12) |  |  |
| Transfer of | | | | |
| Drug history for admitted patients (9) |  |  |  |  |
| **2. Vital signs; clinical examinations; and investigations, tests and procedures** | | | | |
| Clinical examinations | | | | |
| Perform | Offer | Chaperone (EPPC33) |  |  |
|  | Technique | In a dignified manner (EPPC33) |  |  |
|  |  |  |  |  |
| Review findings (22) | Type | Musculoskeletal | Costophrenic angle tenderness (11) |  |
| Investigations | | | | |
| Order/request | Technique | Request only relevant blood tests (EPEQ45) |  |  |
| Review results (12, 22) | Type | Culture | Urine culture (11) |  |
| Tests and procedures | | | | |
| Order / request | Technique | Fewer tests (EPEFFECT26) |  |  |
|  |  | Only necessary tests (EPEFFIC37) |  |  |
|  | Type | Procalcitonin test (EPEFFECT27) |  |  |
| Perform | Technique | Use equipment for appropriate pathway (EPEFFIC38) |  |  |
| Review result | Type | Pregnancy test | Pregnancy status (11) |  |
| **3. Diagnosis** | | | | |
| Diagnose patient | Technique | Appropriately (EPS22) |  |  |
|  | Type | Severe sepsis or septic shock (6) |  |  |
| Educate and train patient | About their diagnosis and condition (EPPC32) |  |  |  |
| **4. Management planning** | | | | |
| Management plan | Involve patient | Provide patient with information about their options (EPPC33) |  |  |
|  | Technique | Respect patient beliefs and decisions (EPPC33) |  |  |
|  | Identify | Patients suitable for ambulation and treatment that suits the service (EPT35) |  |  |
| **5. Treatment in the ED (5)** | | | | |
| General approach | | | | |
| Technique | Treat condition rather than patient (EPEQ21) |  |  |  |
|  | Review patient’s own drugs (1) |  |  |  |
| Prescribe | | | | |
| Initiate prescription chart (18) |  |  |  |  |
| Re-start when appropriate (EPS22) |  |  |  |  |
| Combination products (EPEFFECT30) |  |  |  |  |
| Evidence based medicines (EPEQ44) |  |  |  |  |
| According to: | Policies (EPS23) (EPS24) |  |  |  |
|  | Procedures (EPS23) |  |  |  |
|  | Guidelines (EPS23) |  |  |  |
|  | Formulary (EPS23) |  |  |  |
|  | Biochemical parameters (EPS23) |  |  |  |
|  | Medicines reconciliation (EPS23) |  |  |  |
| Technique | Correctly (EPS22) (EPEFFECT28) |  |  |  |
|  | Appropriately (EPEFFECT28) |  |  |  |
|  | Timely (EPEFFECT29) |  |  |  |
|  | Only if necessary (EPEFFECT30) (EPEFFIC39) |  |  |  |
|  | Assess patients’ personal characteristics (EPEQ42) |  |  |  |
|  | Without bias due to individual factors (EPEQ42) (EPEQ43) |  |  |  |
| Scribe | | | | |
| Drug chart (16) |  |  |  |  |
| Prescription for: | Post-intubation analgesia (14) |  |  |  |
| Calculate dose | | | | |
| For: | Tissue Plasminogen Activator (26, 28) |  |  |  |
|  | Antimicrobials (EPEFFECT5) |  |  |  |
| De-prescribe (EPEFFECT6) (EPEFFECT30) | | | | |
| Reduce unnecessary injections (EPEFFECT30) |  |  |  |  |
| If: | Presenting complaint compromised (EPS4) |  |  |  |
|  | Medicine may cause harm (EPS22) |  |  |  |
| Clinical check of prescription (3, 9, 17, 20, 23) | | | | |
| Technique | Check: | Prescriber adherence to formulary (EPEFFECT7) |  |  |
|  |  | Correct course length (EPS2) | Antimicrobials (EPS2) |  |
|  |  | Adequate course length (EPS2) | Antimicrobials (EPS2) |  |
|  |  | Correct administration times (EPS2) |  |  |
|  |  | There is an indication (EPEFFECT6) |  |  |
|  |  | Appropriate medicines are prescribed (EPEFFECT6) |  |  |
|  |  | Medicines prescribed in accordance with formulary (EPEFFIC17) |  |  |
|  |  | Appropriate formulation prescribed | Child given suspension (EPPC11) |  |
|  |  | For allergies (3) |  |  |
|  |  | Drug interactions (3) |  |  |
|  | Identify: | Critical medicines (EPS2) | Carbamazepine for epilepsy versus pain (EPS2) |  |
|  | Optimise: | Medicines (5, 12) | With respect to cost (EPEFFECT7) |  |
| Prospective | Antimicrobials | For pneumonia (8) |  |  |
|  |  | Check: | Course length (EPEFFECT5) |  |
|  |  |  | Dose (13) |  |
|  |  |  | Dose timings (13) |  |
|  |  |  | Choice (13) |  |
|  |  |  | Interactions (EPEFFECT5) |  |
|  |  |  | For appropriate oral to intra-venous administration switches (EPEFFECT5) |  |
|  |  |  | Compliance with guidelines (EPEFFECT5) |  |
|  | Analgesia post-intubation (14) |  |  |  |
| Retrospective (after patient discharged) | Empirical antimicrobials | For UTI (11) |  |  |
| Identification of: | Contraindications | Recombinant Tissue Plasminogen Activator (26) |  |  |
|  | Interactions (22) (EPS4) |  |  |  |
|  | Adverse drug reaction (13) |  |  |  |
|  | Identify medicines no longer indicated (EPEFFECT30) |  |  |  |
| Early identification of: | Medication related issues (EPEFFECT28) |  |  |  |
| Interventions (17): | Related to opiates: | Stop excessive prescribing (EPEFFECT10) |  |  |
|  |  | Stop prescribing of intra-venous morphine (EPEFFECT10) |  |  |
|  |  | Stop prescribing on patient arrival (EPEFFECT10) |  |  |
|  | Related to Adverse reactions (22) |  |  |  |
|  | Interception of medication error | Early intercept (3) |  |  |
|  |  | Late intercept (3) |  |  |
|  |  | Ameliorated adverse drug event (3) |  |  |
|  |  | Prescribing error (3) (22) |  |  |
|  |  | Transcribing error (3) |  |  |
|  |  | Dispensing error (3) |  |  |
|  |  | Administration error (3) |  |  |
|  |  | Monitoring error (3) | Inadequate monitoring (3) |  |
|  |  | Type: | Wrong patient (3) |  |
|  |  |  | Wrong drug (3) |  |
|  |  |  | Drug omission (3, 32) |  |
|  |  |  | Drug duplication (3) |  |
|  |  |  | Drug class duplication (3) |  |
|  |  |  | Contraindication (3) (EPEFFECT30) |  |
|  |  |  | Patient allergic (3) |  |
|  |  |  | Drug-drug interaction (3) |  |
|  |  |  | Intravenous incompatability (3) |  |
|  |  |  | Wrong form (3) (22) |  |
|  |  |  | Wrong strength (3) |  |
|  |  |  | Wrong route or technique (3) |  |
|  |  |  | Dose omission (3) |  |
|  |  |  | Wrong dose units (22) |  |
|  |  |  | Extra dose (3) |  |
|  |  |  | Underdose (3) |  |
|  |  |  | Overdose (3) |  |
|  |  |  | Rate too fast or slow (3) |  |
|  |  |  | Wrong frequency (3) |  |
|  |  |  | Wrong duration (3) |  |
|  |  |  | Wrong time (3) |  |
|  |  |  | Wrong treatment protocol (3) |  |
|  |  | Concerning: | Antimicrobials (3) |  |
|  |  |  | Central nervous system drugs (3) |  |
|  |  |  | Anticoagulants and thrombolytics (3) |  |
|  |  |  | Cardiovascular drugs (3) |  |
|  |  |  | Hormonal agents (including insulin) (3) |  |
|  | Recommendation to (13): | Discontinue medicine (4, 10) | Due to no indication (22) |  |
|  |  | Optimise dose (4, 10, 22, 23) | Based on renal function (4) |  |
|  |  | Add new medicine (4, 6, 10) | Empirical antibiotics (6) |  |
|  |  |  | Antibiotic (2) |  |
|  |  |  | For indication requiring treatment (22) |  |
|  |  | Change dose frequency (4, 22) |  |  |
|  |  | Change medicine (4, 7, 10, 22, 23) | Asthma medication (2) |  |
|  |  |  | COPD medication (2) |  |
|  |  |  | Antibiotics (2) |  |
|  |  |  | Empirical antibiotics | For Urinary Tract Infection (11) |
|  |  |  | Pain medication (2) |  |
|  |  |  | Anti-emetics (2) |  |
|  |  |  | Reason: | Formulary interchange (10) |
|  |  |  |  | Due to allergy notification (10) |
|  |  | Resume a medicine (4) |  |  |
| Interventions: | | | | |
| Focused on: | Optimisation of medicine use (30) |  |  |  |
|  | Medicines efficacy (30) |  |  |  |
|  | Medicines safety (30) |  |  |  |
|  | Medicines tolerability (30) |  |  |  |
| Identification of: | Medicine absent (EPS2) | Order quickly (EPS4) |  |  |
|  | Medicine prescribed incorrectly (EPEFFECT25) | Prescribe correct medicine (EPEFFECT25) |  |  |
| Identification of: | | | | |
| Appropriate treatment for medication related admissions (EPT34) |  |  |  |  |
| Potential Adverse Drug Reaction (EPS4) | Predict long-term effect (EPS4) |  |  |  |
| Request medicine | | | | |
| Intubation medicines (2) |  |  |  |  |
| Post-intubation medicines (2) |  |  |  |  |
| Cardiac medicines (2) | Nitro-glycerin (2) |  |  |  |
|  | Anti-hypertensives (2) |  |  |  |
| Seizure medicines (2) |  |  |  |  |
| Tissue Plasminogen Activator (26) |  |  |  |  |
| Procure medicine | | | | |
| Empirical antibiotics (6) |  |  |  |  |
| Tissue Plasminogen Activator (26) |  |  |  |  |
| Order medicine (EPS2) | | | | |
| Technique: | Follow-up the order (EPS2) |  |  |  |
|  | Only quantity required (EPEFFIC39) |  |  |  |
| Type: | non-stock medication (EPT15) | Critical medicines (EPT15) |  |  |
| Obtain medicine | | | | |
| Analgesia (14, 19) |  |  |  |  |
| Dispense medicines (19) | | | | |
| Urgent medicines (1) |  |  |  |  |
| Commonly prescribed oral medications (10) |  |  |  |  |
| Prepare necessary I.V. medications (10) |  |  |  |  |
| Supply medicines (1) | | | | |
| Communicate with pharmacy department (EPS2) |  |  |  |  |
| Responding to patients requesting medicines (1) |  |  |  |  |
| Prepare medicine for administration | | | | |
| Tissue Plasminogen Activator (21, 26, 28) |  |  |  |  |
| Pain medication (19) |  |  |  |  |
| Administer medicines | | | | |
| Empirical antibiotics (6) |  |  |  |  |
| Provide advice to healthcare professional (4, 5) | | | | |
| Clinical advice (31) |  |  |  |  |
| Recommendations (3): | Initiate analgesia post-intubation (14) |  |  |  |
|  | Prescribe protective medicines(EPS4) | Proton Pump Inhibitor whilst on NSAIDs (EPS4) |  |  |
|  | Not prescribe unless necessary (EPEFFECT5) |  |  |  |
|  | Related to Evidence Based Medicine (10) |  |  |  |
| Drug advice (10) | Related to: | Rate of drug administration (10) |  |  |
|  |  | Compatibility of intravenous medications (14) |  |  |
|  |  | Dosage (14) | Titration (14) |  |
|  |  | Therapeutic drug monitoring (10) |  |  |
|  |  | Antibiotics | Course length (EPEFFECT5) |  |
|  |  |  | Route (EPEFFECT5) |  |
|  |  |  | Dosage (EPEFFECT5) |  |
|  | Proactive advice related to: | Urgent situation (22) |  |  |
|  |  | Appropriate dose of antibiotics |  |  |
|  |  | Therapeutic suggestions | Verbal (16) |  |
|  |  |  | Written (16) |  |
|  |  | Optimisation of analgesics (19) |  |  |
|  |  | Formulary options (EPEFFECT7) |  |  |
|  |  | Newly prescribed medicine doses | To support prompt administration (EPT36) |  |
|  | Reactive advice related to: | Issues with medicines (EPS1) |  |  |
|  |  | General issues (1) |  |  |
|  |  | Clinical issues (1) |  |  |
|  |  | Ineffective medicine (EPEFFECT6) |  |  |
|  |  | Stock available | Use stock immediately available where possible (EPT16) |  |
| Refer to others | | | | |
| Expert / senior colleague (EPS1) |  |  |  |  |
| Microbiology (EPEFFECT5) |  |  |  |  |
| Review: | | | | |
| Medication (EPEFFECT6) (EPEFFECT8) |  |  |  |  |
| Offer: | | | | |
| Preventative care | Vaccination (5) | Flu (5) |  |  |
| Respond to: | | | | |
| Toxicological emergencies (5) |  |  |  |  |
| Participation in: | | | | |
| Resuscitation: | Sepsis resuscitation (27) |  |  |  |
|  | Trauma resuscitation (3) |  |  |  |
|  | Arrest resuscitation (3) |  |  |  |
| Procedures | | | | |
| Perform procedure: | Vaccination (5) | Flu (5) |  |  |
|  | Counsel patient about procedure: | Flu vaccination (5) |  |  |
|  | Appropriate procedure (EPEFFECT28) |  |  |  |
| Remind doctor of procedure: | Vaccination | Tetanus (2) |  |  |
|  |  | Diphtheria (2) |  |  |
| Follow Trust policies (EPS24) |  |  |  |  |
| Patient counselling | | | | |
| Communicate: | Side effects (EPS4) |  |  |  |
|  | Why no treatment has been prescribed (EPEFFECT6) |  |  |  |
|  | Information about treatment (EPEFFECT8) | How to use e.g. inhaler (EPEFFECT8) |  |  |
|  |  | Why given (EPPC12) |  |  |
| Educate on: | Use of newly prescribed medicines (EPS22) |  |  |  |
|  | How the treatment will work (EPPC32) |  |  |  |
|  | Best time to take the medication (EPPC32) |  |  |  |
|  | Common side effects (EPPC32) |  |  |  |
|  | Why treatment is appropriate for condition (EPPC32) |  |  |  |
| Check: | Appropriate use (EPEFFECT8) |  |  |  |
|  | Elicit patient’s knowledge and views of their treatment (EPPC13) |  |  |  |
| Involve: | In decisions (EPEFFECT8) | Offer choice of therapies (EPPC12) |  |  |
| Explore: | Expectations (EPS22) |  |  |  |
| Resolve: | Issue(s) (EPS22) |  |  |  |
| **6. Monitoring** | | | | |
| Overall and general review (1, 4) |  |  |  |  |
| Therapeutic drug monitoring (EPS4) |  |  |  |  |
| Vital signs | | | | |
| Review | Clinical signs and symptoms of infection (7) |  |  |  |
| Clinical examination | | | | |
| Perform: | Body examination (e.g. external body) (5) |  |  |  |
| Investigations | | | | |
| Order / request: | Vancomycin bloods at the correct time (EPEFFECT26) |  |  |  |
| Recommend (13) |  |  |  |  |
| Review the results of (3): | Clinical signs and symptoms of infection (7) |  |  |  |
| Tests and procedures | | | | |
| Review the results of: | Urinalysis (7) |  |  |  |
|  | Culture and susceptibility reports (7) (EPEFFECT5) |  |  |  |
| Ensure that: | Blood culture samples drawn before antibiotic administration (13) |  |  |  |
| Diagnosis | | | | |
| Review diagnosis (7) |  |  |  |  |
| Follow-up patient after discharge | | | | |
| To review: | Cultures and discharge antimicrobials prescribed (29) | Recommendations made to mid-level provider | No change to prescribed antimicrobial (15) |  |
|  |  |  | Discontinue antimicrobial (15) |  |
|  |  |  | Modify antimicrobial (15) |  |
|  |  |  | Discontinue and modify if symptoms occur (15) |  |
|  |  |  | Start new antimicrobial (15) |  |
|  |  |  | Start new antibiotic if symptoms occur (15) |  |
|  | Appropriateness of discharge treatment (7) |  |  |  |
| To recommend: | Treatment modification (11, 29) |  |  |  |
|  | Treatment initiation (29) |  |  |  |
| **7. Discharge / admission to hospital** | | | | |
| Discharge | | | | |
| Treatment | Prescribe medicines (EPEFFECT28) |  |  |  |
|  |  | Transcribe outpatient or TTO prescriptions (EPT14) |  |  |
|  | Clinical check of prescription (7, 20, 31) |  |  |  |
|  | Dispense medicines | In the ED (EPT14) |  |  |
|  | Supply medicines (1) |  |  |  |
|  | Counsel | Medicines focused (4, 5, 12) |  |  |
|  |  | Condition focused |  |  |
|  |  | Technique: | Ensure patient given correct advice (EPEFFECT28) |  |
|  | Interventions related to: | Optimisation of medicine use (30) |  |  |
|  |  | Medicine efficacy (30) |  |  |
|  |  | Medicine safety (30) |  |  |
|  |  | Tolerability of treatment (30) |  |  |
|  | Refer to: | Community pharmacy | For prescription (7, 11) |  |
|  |  | Community services (EPEFFECT28) |  |  |
|  |  | Technique: | Appropriately (EPS22) (EPT35) |  |
|  | Communication with Primary Care (EPS22) | Recommendations related to: | Future management (12) |  |
|  |  |  | Patient-care (12) |  |
|  |  |  | Medicines issues (12) |  |
| Write discharge letter (EPEFFECT28) |  |  |  |  |
| Admission to hospital | | | | |
| Write inpatient drug chart (1) |  |  |  |  |
| Transfer medicines | Patients own drugs (1) |  |  |  |
|  | Technique: | Ensure transfer of medicines |  |  |

Table 4. Processes: General

| **Level 1 Process** | **Level 2 Process** | **Level 3 Process** | **Level 4 Process** |
| --- | --- | --- | --- |
| Develop: | Guideline(s) (1) (EPEFFECT9) (EPS23) | Integrated guidelines for: | Critical care intra-venous medicines (EPEFFECT29) |
|  | Policies (EPEFFECT9) (EPS24) |  |  |
|  | Procedures (EPEFFECT9) |  |  |
|  |  | Technique: | Using evidence from therapeutic review (EPEFFECT9) |
|  | Patient Group Directions (1) |  |  |
|  | Checklists (EPEFFIC37) |  |  |
| Review: | Guideline(s) (1) |  |  |
|  | Policies (EPS24) |  |  |
|  | Procedures (EPS24) |  |  |
|  | Patient Group Directions (1) |  |  |
|  | Adherence to: | Guideline(s) (3) |  |
|  |  | Protocol(s) (3) |  |
|  | Ward stock (EPEFFIC19) |  |  |
| Share: | Policies (EPEFFIC40) |  |  |
| Collate: | Ideas about ED pharmacist practice (EPEFFIC40) |  |  |
| Standardise: | Care across EDs (EPEFFIC40) |  |  |
| Answer questions related to: | Post-intubation analgesia (14) |  |  |
| Participate in: | Discussion about post-intubation analgesia (14) |  |  |
|  | Department rounds (3) |  |  |
| Educate and train (EPEFFECT9) (EPS24) | Doctors about (1, 7): | Post-intubation analgesia (14) |  |
|  |  | Community Acquired Pneumonia | Appropriate antibiotic selection (33) |
|  |  |  | Appropriate antibiotic administration times (33) |
|  |  |  | Use of prescription pro-forma (33) |
|  |  | The role of the ED pharmacist (33) |  |
|  |  | Use of Antibiogram for Urinary Tract Infections (24) |  |
|  |  | Correct use of antibiotics (34) |  |
|  | Nurses about (1): | Post-intubation analgesia (14) |  |
|  |  | Community Acquired Pneumonia | Appropriate antibiotic selection (33) |
|  |  |  | Appropriate antibiotic administration times (33) |
|  |  |  | Use of prescription pro-forma (33) |
|  |  | The role of the ED pharmacist (33) |  |
|  | Prescribers | To support resolution of medicines queries (ESP1) |  |
|  | Other healthcare professionals about: | Bioavailability (EPEFFECT26) |  |
| Raise staff awareness of (EPS24): | Guidelines |  |  |
| Support: | Staff induction training (1) |  |  |
|  | Risk management (1) |  |  |
|  | Finances: | Medicines expenditure (1) |  |
|  |  | Medicines budget (1) |  |
| Advise: | Patients bring their own drugs from home through the ‘patient’s own drugs’ campaign (EPEFFIC18) |  |  |
| Vet: | Drug company representatives (EPEQ43) |  |  |

To increase usability of the framework, outcome indicators have been categorised as either: quantitative (green); qualitative (yellow); quantitative and/or qualitative (blue) and those which lacked specificity (red). The latter mainly includes suggestions from experts which more closely resembled themes or ideas rather than indicators that could be measured. These have been included as they could be further developed/defined for future evaluation purposes.

Table 5. Outcomes and outcome indicators: Safe care

| **Outcomes** | **Outcome indicators: measure…** | **Data source?** |
| --- | --- | --- |
| Safety of influenza vaccination service (5) | Number of patients who experienced an adverse outcome (5) | - Hospital incident reports - Patient surveys |
|  | Number of adverse incidents (5) |  |
|  | Review types of adverse incidents (5) |  |
|  | Patient experience of having the vaccination e.g. any adverse events (MTDEP) |  |
| Medication error (with or without consequence to patient) (2) | Number of patients who received a medication error (2) | - Hospital incident reports - Yellow-card reports and those who complete them |
|  | Errors of high risk medication e.g. vancomycin (MTDEP) |  |
| Harmful medication error (2) | Number of patients who were harmed due to medication error (2) | - Hospital incident reports - Observation data - Medical notes and drug charts - Serious Adverse Event Reports |
|  | Length of stay (MTDEP) |  |
|  | Clinical outcomes (MTDEP) |  |
| Discontinuation of analgesia (14) | Number of patients where analgesic therapy was discontinued due to adverse incident (14) | - Medical notes - Discharge information related to discontinued medicines - Pain scores - Function scores |
|  | Time before re-starting analgesia (MTDEP) |  |
|  | Whether analgesia was stopped before their visit to the ED (MTDEP) |  |
| Pharmacist prescribing safety (18) | Number of prescribing errors (18) | - Those with responsibility for governance of non-medical prescribers - Drug charts |
|  | Number of prescribing errors per patient (18) |  |
|  | Re-attendance rates to ED and GP due to a specific drug prescribed (MTDEP) |  |
|  | Reasons for re-attendance to ED and GP (MTDEP) |  |
|  | Follow-up with GP patients who pharmacists prescribed for (MTDEP) |  |
|  | Prescribing versus allergy status (MTDEP) |  |
| Resolve medicines queries (EPS1) | Review staff feedback about response to queries (EP) (MTDEP) | - Staff |
|  | Review local data (EP) |  |
|  | Evaluate local interventions (EP) |  |
|  | Time from query to the response from the pharmacist (MTDEP) |  |
|  | Review queries 48 hours after they were made to see if resolved (EP) |  |
|  | Review unresolved queries (EP) |  |
|  | Patient satisfaction with response to query (MTDEP) |  |
|  | Time taken to resolve query (MTDEP) |  |
| Timely supply of medication (EPS2) | Time from stock item prescribed to administration (EP) | - Stock levels of important medicines |
|  | Time from non-stock item prescribed to administration (EP) |  |
|  | Number of missed doses (MTDEP) |  |
|  | Evaluate local missed doses (EP) |  |
|  | Time to first dose of antibiotic given by nurses e.g. in sepsis patients (MTDEP) |  |
|  | Time medicine requested to time of delivery (EP) |  |
|  | Time medicine requested to time of accuracy check (EP) |  |
|  | Review delays in medication administration times (EP) |  |
|  | Number of doses given because of patients missing a dose as prescribed e.g. Parkinson’s patients (MTDEP) |  |
|  | Time to supply of critical, urgent and non-urgent medicines (MTDEP) |  |
|  | Review missed doses due to drugs not available (EP) |  |
| Accurate medication history / medicines reconciliation (EPS3) | Evaluate incident reports associated with incorrect medication history (EP) | - Summary care reports - Local systems |
|  | Number of errors in medication history / medicines reconciliation (EP) |  |
|  | Whether medicines reconciliation was undertaken (MTDEP) |  |
|  | Medications given in ED compared with those recorded in medication history / medicines reconciliation (MTDEP) |  |
|  | Comparison of medicines reconciliation with optimal medicines reconciliation (MTDEP) |  |
|  | Time to gather the correct information from different sources (MTDEP) |  |
|  | Whether critical medicines are prescribed (MTDEP) |  |
|  | Evaluate errors in medication history / medicines reconciliation (EP) |  |
| Adverse drug events (EPS4) | Evaluate pharmacist interaction with patient or advice given (EP) | - Local systems (flagged Adverse Drug Events) - Observations - Clinical presentation of patients - Incident reports - Clinical outcomes - Lab results to identify Adverse Drug Events |
|  | Number of incidents reported (EP) |  |
|  | Number of yellow cards submitted (EP) |  |
|  | Severity of Adverse Drug Reactions (MTDEP) |  |
|  | Number of patients admitted with specific keywords in presenting complaint (EP) |  |
|  | Evaluate Datix reports (EP) |  |
| Prevention of re-attendance (EPS22) | Patient re-attendance ≤72 hours (EP) | - Hospital records - GP records - Multidisciplinary meetings in the community (especially drug and alcohol users) |
|  | Number of patients who re-attend ≤7 days with same presenting complaint (EP) |  |
|  | Unplanned attendance to GP practice ≤7 days (EP) |  |
|  | GP follow-ups (MTDEP) |  |
|  | Community pharmacy follow-ups (MTDEP) |  |
|  | Patient re-attendance at ED with same presenting complaint as original attendance, or a sequelae of this, within time-frame specific to condition (EP) |  |
|  | Number of re-admissions in an appropriate period (EP) |  |
|  | Compare post-discharge care with care planned (MTDEP) |  |
|  | Re-attendance rate comparison by disciplines (MTDEP) |  |
| Prescribing errors (EPS23) | Number of adverse incidents for pharmacist prescriptions (EP) | - Hospital / other incident reports - Medical notes - Pharmacist intervention record - Near miss records |
|  | Number of interventions made for pharmacist prescriptions (EP) |  |
|  | Review local incident reports that concern prescribing errors (EP) |  |
|  | Evaluate types of prescribing error (EP) |  |
|  | Number of prescribing errors (MTDEP) |  |
|  | When prescribing errors are made (MTDEP) |  |
|  | How prescribing errors were resolved (MTDEP) |  |
|  | Extent to which pharmacists prevent errors from reaching the patient (MTDEP) |  |
| Treatment which adheres to Trust policy and guidelines (EPS24) | Evaluate prescribing practices for inpatients (EP) |  |
|  | Evaluate prescribing practices for outpatients (EP) |  |
|  | Prescriber adherence to empirical treatment guidelines by condition (EP) |  |
|  | Adherence to local/national antimicrobial guidelines and policies (EP) (MTDEP) |  |
|  | Impact of treatment advised by pharmacist (EP) |  |
|  | Impact of pharmacist teaching in the ED (EP) |  |
|  | Awareness of policies (EP) |  |

Table 6. Direct outcomes and outcome indicators: Effective care

| **Outcomes** | **Outcome indicators: measure…** | **Data source?** |
| --- | --- | --- |
| Feasibility of influenza vaccination service (5) | Time required to screen patients (5) |  |
|  | Time required to counsel patients (5) |  |
|  | Time required to Vaccinate patients (5) |  |
|  | Number of vaccinations administered (5) |  |
|  | Number of patients requesting influenza vaccination (MTDEP) |  |
| Appropriateness of antimicrobial treatment in patients with sepsis or septic shock (6) | Number of patients who receive appropriate antimicrobials (6) |  |
|  | Antimicrobial stewardship activities (MTDEP) |  |
|  | Antimicrobial treatment given against blood cultures (MTDEP) |  |
|  | Whether practitioner seeing patient is appropriate (MTDEP) |  |
|  | Adherence to local/national guidelines and policies (MTDEP) |  |
| Value of an antimicrobial stewardship service (7) | Number of pharmacist interventions for inappropriate therapy (7) | - Opinion poll |
|  | Time between discharge and when contact made after discharge (7) |  |
|  | Awareness of antimicrobial stewardship policy (MTDEP) |  |
|  | Length of stay (MTDEP) |  |
| Reduction of patient blood pressure to <185/110mmHg for Tissue Plasminogen Activator treatment (26) | Number of patients who were prescribed less-than ideal anti-hypertensives (26) |  |
|  | Number of patients where labetalol dose escalation failed to adequately lower blood pressure in a timely manner and nicardipine IV infusion was used (26) |  |
|  | Whether reduction of blood pressure to <185/110mmHg is achieved (26) |  |
| Appropriateness of revised or newly prescribed antimicrobials (29) | Number of inappropriately revised antimicrobials (29) | - Patient’s pathway |
|  | Number of inappropriate newly prescribed antimicrobials (29) |  |
|  | Adherence to local/national antimicrobial guidelines and policies (MTDEP) |  |
|  | Patient scores (MTDEP) |  |
|  | Patient improvement (MTDEP) |  |
|  | Number of viral infections treated with antibiotics (MTDEP) |  |
|  | Timeframe of antimicrobials prescribed (MTDEP) |  |
| Sepsis related mortality (27) | Number of patients with sepsis (27) | - Mortality rates (hospital dataset) |
|  | Number of patients with sepsis who died (MTDEP) |  |
|  | Number of patients with severe sepsis who died in hospital (27) |  |
|  | Number of patients with septic shock who died in hospital (27) |  |
| Sepsis criteria (27) | Appropriateness of antimicrobial used to treat Sepsis (27) | - Standardised sepsis audit tool |
|  | Adherence to guidelines (MTDEP) |  |
|  | Number of clinical check interventions (27) |  |
|  | Type of clinical check interventions (27) |  |
| Appropriate use of Primary Care services by patients after discharge (12) | Number of visits to Primary Care providers per patient (12) (MTDEP) | - Friends and family test - Primary care dataset - Feedback from primary care - Patient feedback - GP appointment records |
|  | Number of visits to Urgent Care providers per patient (12) |  |
|  | Number of visits to ED per patient (12) |  |
|  | Number of visits to ED in 90 days prior to current visit (12) |  |
|  | Number of visits to ED 1-30 days after discharge (12) |  |
|  | Number of visits to ED 31-60 days after discharge (12) |  |
|  | Number of visits to ED 61-90 days after this visit (12) |  |
|  | Number of patients seeking GP advice (MTDEP) |  |
|  | Number of patients seeking advice from NHS 111 (MTDEP) |  |
|  | Types of services being used (MTDEP) |  |
|  | Opinions of primary care service providers as to whether their services were used appropriately (MTDEP) |  |
| Initiation of post-intubation analgesia (14) | Number of patients who received post-intubation analgesia (14) (MTDEP) | - Medication records - Sedation records - Medical notes - Drug chart - Medical notes |
|  | Time between intubation and analgesia commenced (MTDEP) |  |
|  | Current practice against policy/guidelines (MTDEP) |  |
| Use of sedative or anxiolytic after intubation without analgesia (14) | Number of patients who received sedative or anxiolytic therapy after intubation but without analgesia (14) (MTDEP) | - Drug chart - Medical notes |
| Effective use of antibiotics in patients with potential Urinary Tract Infections (15) | Number of days of antimicrobial therapy potentially avoided (15) | - Patient prescription - Patient feedback data - Sensitivities from microbiology |
|  | Number of patients with potential Urinary Tract Infections for whom antibiotics were commenced (MTDEP) |  |
|  | Antibiotic use against local/national guidelines (MTDEP) |  |
|  | Whether infection improving (MTDEP) |  |
|  | Clinical outcomes (MTDEP) |  |
|  | Number of re-attendances to GP/ED (MTDEP) |  |
| Effective use of analgesia in trauma patients (19) | Change in pain score from arrival at hospital to transfer to ED (19) | - Patient notes - Visual Analogue Scale for pain - Pain scores - Trauma scoring systems |
|  | Change in pain score from arrival at hospital to transfer to ED in patients who were given analgesia by the ambulance service throughout transfer (19) |  |
|  | Change in pain score from arrival at hospital to transfer to ED in patients who were not given analgesia by the ambulance service throughout transfer (19) |  |
|  | Change in pain score before and after analgesia administered (MTDEP) |  |
|  | Whether patients receive analgesia in a timely manner (MTDEP) |  |
|  | Whether patients receive analgesia appropriate to their injuries (MTDEP) |  |
|  | Number of patients receiving adequate analgesia (MTDEP) |  |
|  | Time from arrival at ED to first analgesia (MTDEP) |  |
|  | Time to first assessment (MTDEP) |  |
| Antibiotic resistance (EPEFFECT5) | Prescriber adherence to protocols or guidelines for empirical treatment of conditions (EP) | - Blood cultures - Patient experience data - Long-term clinical outcomes |
|  | Prescriber adherence to protocols or guidelines for empirical treatment of conditions at discharge (EP) |  |
|  | Resistance patterns over time (EP) |  |
| Unnecessary prescribing (EPEFFECT6) | Review incident reports that relate to incorrect prescribing (EP) | - Patient medication record - Drug chart - Ward stock - Observation data - Staff discussion - Finance reports |
|  | Adherence to national prescribing guidelines and protocols (EP) |  |
|  | Evaluate whether ‘unnecessary’ medicines are prescribed (EP) (MTDEP) |  |
|  | Whether medicines prescribed were given / taken (MTDEP) |  |
|  | Evaluate de-prescribing (EP) |  |
|  | Number of medications the pharmacist advise should be stopped (EP) |  |
|  | Number of medications wasted (MTDEP) |  |
|  | Excessive spend on medicines (MTDEP) |  |
|  | Staff opinion (MTDEP) |  |
| Cost effective prescribing (EPEFFECT7) | Drug expenditure (EP) | - Patient medication record - British National Formulary - Observation data - Staff discussion - Finance reports |
|  | Drug preparation time (EP) |  |
|  | Stock holding (EP) |  |
|  | Review ‘Horizon Scanning’ (EP) |  |
|  | Review budget management (EP) |  |
|  | Number of non-formulary drug requests (EP) |  |
|  | Expenditure on specific drugs (EP) |  |
|  | Whether drug prescribed is cheapest available (MTDEP) |  |
|  | Excessive spend on medicines (MTDEP) |  |
|  | Staff opinion (MTDEP) |  |
| Effective use of treatment by patient (EPEFFECT8) | Patient knowledge of condition (EP) | - Patient feedback - Patient follow-up data - Clinical outcomes |
|  | Patient knowledge of treatment (EP) |  |
|  | Patient re-attendance at ED with same presenting complaint as original attendance, or a sequelae of this, within time-frame specific to condition (EP) (MTDEP) |  |
|  | Number of admissions due to poor use of medicines (EP) |  |
|  | Review how patient uses their medicine (EP) |  |
|  | Patient compliance (MTDEP) |  |
|  | Review patient’s self-care (e.g. use of medicines) prior to hospital (MTDEP) |  |
|  | Clinical outcomes (MTDEP) |  |
| Evidence based policies and procedures (EPEFFECT9) | Review pharmacist involvement in guideline development, implementation and audit (EP) |  |
|  | Review pharmacist involvement in policy development and production (MTDEP) |  |
|  | Pharmacist involvement in clinical governance meetings (MTDEP) |  |
|  | Review pharmacist involvement in teaching (EP) |  |
|  | Review the evidence used to develop policies (EP) |  |
|  | Review local documents and compare these with national guidance (EP) |  |
| Adequate and appropriate analgesia (EPEFFECT10) | Change in pain scores (EP) (MTDEP) |  |
|  | Rate of change in pain scores (MTDEP) |  |
|  | Reduction of pain scores (MTDEP) |  |
|  | Change in function scores (MTDEP) |  |
|  | Review pharmacist involvement in teaching (EP) |  |
|  | Time to first dose (EP) (MTDEP) |  |
| Appropriateness of prescribing (EPEFFECT25) | Number of interventions required for pharmacist prescription (EP) |  |
|  | Number of amendments made to original pharmacist prescription (EP) |  |
|  | Adherence to local/national guidelines (MTDEP) |  |
|  | Evaluate missed doses (EP) |  |
|  | Review prescribing error incident data (EP) |  |
|  | Review interventions (EP) |  |
| Number of invasive procedures (EPEFFECT26) | Number of cannulations (EP) |  |
|  | Number of tetanus vaccinations compared with guidance (EP) |  |
|  | Evaluate vancomycin prescribing (EP) |  |
|  | Evaluate appropriate use of antimicrobials and vaccinations for wound prophylaxis (EP) |  |
|  | Review pharmacist involvement in development or review of wound guidelines (EP) |  |
|  | Review pharmacist involvement in teaching pharmacokinetics or pharmacodynamics (EP) |  |
|  | Number of vancomycin levels undertaken (EP) |  |
| Antimicrobial resistance (EPEFFECT27) | Number of antimicrobial prescriptions avoided by use of procalcitonin test (EP) | - Long-term outcomes data |
|  | Review of national data (EP) |  |
|  | Evaluate resistance to different antibiotics for specific infections (EP) |  |
|  | Review local antimicrobial data (EP) (MTDEP) |  |
|  | Recurrent infections (MTDEP) |  |
| Reduce length of stay (EPEFFECT28) | Length of stay for specific clinical groups (EP) |  |
|  | Time to diagnosis (EP) |  |
|  | Time to admission (MTDEP) |  |
|  | Length of hospital stay (EP) |  |
|  | Review length of stay data for specific cohorts or areas of hospital (EP) |  |
|  | Review impact of early consideration of chronic disease on overall outcome (EP) |  |
|  | Discharge medication delays (MTDEP) |  |
|  | Time to deliver discharge medicines (MTDEP) |  |
| Reduce mortality (EPEFFECT29) | Mortality data for patients of different acuity (EP) |  |
|  | Morbidity data for patients of different acuity (EP) |  |
|  | Medication related ‘avoidable deaths’ (EP) |  |
|  | Review pharmacist contribution to complex guidelines (EP) |  |
|  | Number of patient deaths (EP) |  |
|  | Number of patient deaths where pharmacists intervened versus when they did not (MTDEP) |  |
|  | Review local data or statistics (EP) |  |
| Reduce polypharmacy (EPEFFECT30) | Number of medicines de-prescribed (EP) |  |
|  | Number of medicines acutely de-prescribed (EP) |  |
|  | Number of medicines patient’s prescribed upon arrival in the ED compared to number of medicines patients were prescribed when discharged (MTDEP) |  |
|  | Number of patients with polypharmacy upon arrival in the ED compared to number of patients with polypharmacy when discharged (MTDEP) |  |
|  | Number of medicines initiated for a particular clinical cohort (EP) |  |
|  | Evaluate interventions made for polypharmacy patients (EP) |  |
|  | Evaluate pharmacist de-prescribing (EP) |  |
|  | Evaluate medicines optimisation undertaken by pharmacist (EP) |  |
|  | Evaluate brief interventions made (EP) |  |
|  | Evaluate pharmacist referrals for a Medicines Use Review or General Practitioner review (EP) |  |

Table 7. Direct outcomes and outcome indicators: Patient centred care

| **Outcomes** | **Outcome indicators: measure…** |  |
| --- | --- | --- |
| Patient satisfaction at discharge (30) | Number of patients dissatisfied with their visit after discharge (30) | - Focus groups / interviews with patients - Patient satisfaction surveys - Complaints |
|  | Patient’s understanding at discharge (30) |  |
| Appropriate medicine formulation (EPPC11) | Adherence to local/national guidelines (EP) |  |
|  | Pharmacist prescribing of different formulations (MTDEP) |  |
|  | Evaluate missed doses where patient unable to swallow (EP) |  |
|  | Evaluate missed doses where patient refused medicine (EP) |  |
|  | Patient opinion of formulation given (EP) |  |
|  | Specific patient’s opinions of formulation given (e.g. those with nasogastric tubes, children, stroke patients) (MTDEP) |  |
|  | Number of liquid preparations given, or other formulations (MTDEP) |  |
|  | Evaluate patient choking (EP) |  |
|  | Compare formulation given with formulary (EP) |  |
| Patient preference of therapy (EPPC12) | Evaluate patient-centred decision making (EP) | - Clinical pharmacist patient satisfaction survey |
|  | Patient satisfaction (EP) |  |
|  | Patient knowledge of therapy (EP) (MTDEP) |  |
|  | Patient contribution to therapy decision (EP) |  |
|  | Time taken to educate patients on therapy options (MTDEP) |  |
| Patient involvement in decision making (EPPC13) | Patient satisfaction (EP) | - Friends and family test - E-mail or text satisfaction data - Clinical pharmacist patient satisfaction survey - Patient satisfaction survey |
|  | Practitioner competency (EP) |  |
|  | Patient contribution to decision (EP) |  |
| Patient satisfaction with consultation (EPPC32) | Patient satisfaction (EP) | - Friends and family test - E-mail or text satisfaction data - Clinical pharmacist patient satisfaction survey - Patient satisfaction survey |
| Patient education and training (EPPC32) | Patient understanding (EP) | - Friends and family test - Clinical pharmacist patient satisfaction survey |
|  | Patient understanding of specific medicines (MTDEP) |  |
|  | Patient compliance after initiation of treatment (EP) |  |
|  | Medication related re-attendance at ED or attendance at General Practice (EP) |  |
|  | Number of patient problems with specific medicines (EP) |  |
|  | Review patient feedback on being educated and trained on their condition (EP) |  |
| Patient respect and dignity maintained (EPPC33) | Evaluate adherence to safeguarding policy (EP) | - Friends and family test - Clinical pharmacist patient satisfaction survey - Patient feedback data |
|  | Review Datix (EP) |  |
|  | Patient satisfaction (EP) |  |
|  | Review pharmacist portfolio with respect to learning about governance (EP) |  |

Table 8. Direct outcomes and outcome indicators: Timely care

| **Outcomes** | **Outcome indicators: measure…** |  |
| --- | --- | --- |
| Time to antibiotic treatment in patients with pneumonia (8) | Time from arrival at ED to antibiotics being prescribed (MTDEP) | - Medication administration record - Patient notes |
|  | Time to first assessment for patients presenting with shortness of breath / chest pain (MTDEP) |  |
|  | Time taken from diagnosis to prescribing to administration (MTDEP) |  |
|  | Time from prescription written to administration (8) |  |
| Time to antimicrobials in patients with severe sepsis or septic shock (6) | Time from arrival at ED to antibiotics being prescribed (MTDEP) | - Medication administration record - Clinical outcomes - Patient notes |
|  | Time taken from diagnosis to prescribing to administration (MTDEP) |  |
|  | Time from prescription written to administration (MTDEP) |  |
|  | Time from patient placement in allocated bay to administration of appropriate antimicrobial (6) |  |
|  | Number of patients for whom appropriate antimicrobials were administered within 3 hours of placement in allocated bay (6) |  |
|  | Time taken to change discharge antimicrobial therapy from inappropriate antimicrobial therapy given in the ED to appropriate antimicrobial therapy (7) |  |
| Time to first dose of antimicrobials in patients with sepsis, severe sepsis or septic shock (27) | Time from identification of sepsis or septic shock to the first dose of antimicrobials (27) | - Medication administration record |
|  | Time from arrival at ED to antibiotics being prescribed (MTDEP) |  |
|  | Time from prescription written to administration (MTDEP) |  |
|  | Time taken from diagnosis to prescribing to administration (MTDEP) |  |
| Review of ‘Sepsis Criteria’ (27) | Time from identification of sepsis to antimicrobial prescription written (27) |  |
|  | Time from antimicrobial prescription written to administration (27) |  |
|  | Whether Sepsis Six was identified on admission (MTDEP) |  |
|  | Time taken for sepsis criteria to be identified (MTDEP) |  |
|  | Compliance with Sepsis 6 within 1 hour of arrival at ED (MTDEP) |  |
|  | Current practice against local/national guidelines (MTDEP) |  |
| Time to administration of Tissue Plasminogen Activator (26) (28) | Time from patient arrival in ED to Tissue Plasminogen Activator administration (26) (MTDEP) | - Medication administration record - Clinical notes |
|  | Number of patients with time from arrival in the ED to Tissue Plasminogen Activator administration of <60 minutes (26) |  |
|  | Time from Computed Tomography scan results to request for Tissue Plasminogen Activator received by pharmacy (28) |  |
|  | Time from Computed Tomography scan results to administration of Tissue Plasminogen Activator (28) |  |
|  | Impact of no illegible orders on time to administration (28) |  |
|  | Impact of no orders with omissions on time to administration (28) |  |
|  | Time from patient arrival in ED to administration of Tissue Plasminogen Activator (21) |  |
|  | Time from decision to administer Tissue Plasminogen Activator to administration of Tissue Plasminogen Activator (21) |  |
|  | Time taken from diagnosis to prescribing to administration (MTDEP) |  |
| Time to initiation of post-intubation analgesia (14) | Time to initiation of post-intubation analgesia (14) | - Medication administration record - Critical care notes |
|  | Time from diagnosis to prescribing to administration (MTDEP) |  |
| Time to administration of pain medication (19) | Time form patient placement in trauma bay to administration of analgesia (19) | - Medication administration record - Medical notes - Patient feedback |
|  | Time from administration of analgesia by Ambulance Service to the first pain medication administered in trauma bay (19) |  |
|  | Time from prescription written to administration (MTDEP) |  |
|  | Time taken from diagnosis to prescribing to administration (MTDEP) |  |
|  | Patient opinion of time taken to retrieve pain medications (MTDEP) |  |
| Time to retrieve pain medications (19) | Time from the retrieval of pain medicines from the ED medicine store to administration to patient (19) | - Visual Analogue Scale for pain |
|  | Time from prescription written to administration (MTDEP) |  |
|  | Patient opinion of time taken to retrieve pain medications (MTDEP) |  |
| Time in department (EPT14) | Patient wait times (EP) | - ED clinical systems i.e. Symphony |
|  | Total time spent in ED (MTDEP) |  |
|  | Time to first assessment (MTDEP) |  |
|  | Time from ambulance handover to when patient leaves department (MTDEP) |  |
| Missed doses due to untimely care (EPT15) | Number of missed doses (EP) | - Patient notes |
|  | Delays in receipt of dose (MTDEP) |  |
| Time to drug available (EPT16) | Time from prescription to administration of stock drug (EP) | - Ordering system - Medication administration record - Datix incident reports related to drugs being unavailable - Observation data |
|  | Time from prescription to administration of non-stock drug (EP) |  |
|  | Whether medicines not available straight away have been ordered by the clinician responsible (MTDEP) |  |
|  | Ward stock levels (MTDEP) |  |
|  | Utilisation of nursing personnel to find available drugs (MTDEP) |  |
| Length of bed stay (EPT34) | Length of stay for specific patient cohort (EP) | - Central hospital bed availability data |
|  | Length of stay in different departments i.e. ED, assessment unit, medical ward (MTDEP) |  |
|  | Length of stay for patients with medication related admission (EP) |  |
|  | Admission avoidance (MTDEP) |  |
| Admissions to hospital (EPT35) | Number of admissions (EP) (MTDEP) | - Admission records - Systems for non-inpatient areas e.g. ambulatory care |
|  | Number of admissions due to medication (MTDEP) |  |
|  | Number of attendances to ambulatory clinics / services (EP) (MTDEP) |  |
|  | Number of patients signposted to other services who re-attend the ED (EP) |  |
|  | Evaluate quality of pharmacist referrals to specialists (EP) |  |
|  | Evaluate pharmacist referrals to community services (EP) |  |
|  | Evaluate pharmacist referrals to General Practitioner (EP) |  |
|  | Evaluate pharmacist referrals to community pharmacy for Medicines Use Review (EP) |  |
|  | Clinical outcomes (MTDEP) |  |
| Time to drug administered (EPT36) | Time from prescription written to nurse informed to medicine administered (EP) | - Medication administration record - Medical notes |
|  | Time from prescription written to medicine administered (EP) (MTDEP) |  |
|  | Evaluate missed doses (EP) |  |
|  | Review delays in administration (EP) |  |
| Timeliness of influenza vaccination service (5) | Time taken to screen patients for eligibility (5) | - Staff discussion - Occupational health records |
|  | Time taken to administer influenza vaccination (5) |  |
|  | Number of patients vaccinated in a given timeframe (MTDEP) |  |
|  | Time taken to vaccinate patients from advertisement to administration (MTDEP) |  |
|  | Number of staff vaccinated (MTDEP) |  |

Table 9. Direct outcomes and outcome indicators: Efficient care

| **Outcomes** | **Outcome indicators** |  |
| --- | --- | --- |
| Suitability of formulation (EPEFFIC17) | Evaluation of prescribing practice (EP) |  |
|  | Evaluate missed doses where patient unable to swallow (EP) |  |
|  | Whether the patient has functional ability required to take the medicine (MTDEP) |  |
|  | Whether drug stock of alternative formulations is borrowed from other wards (MTDEP) |  |
| Use of ‘Patients Own Drug’s’ (POD’s) (EPEFFIC18) | Review drug expenditure on regular medication compared with acute medication (EP) | - Drug charts - POD records - Blister packs brought into ED |
|  | Number of patients using POD’s on ward who had these with them in the ED (EP) |  |
|  | Rate of patient’s repeat prescriptions in community (MTDEP) |  |
|  | Whether patients had PODs with them (MTDEP) |  |
|  | Number of patients using PODs in the ED (MTDEP) |  |
|  | Result of PODs use in the ED (MTDEP) |  |
| Ward stock availability (EPEFFIC19) | Time from prescription written for ward stock item to administration (EP) | - Assistant Technical Officer re-order sheets |
|  | Number of calls to pharmacy by ED (MTDEP) |  |
|  | What is requested from pharmacy (MTDEP) |  |
|  | What is borrowed from other departments / wards (MTDEP) |  |
|  | Evaluate missed doses (EP) |  |
|  | Review (EP) |  |
| Efficiency of care (EPEFFIC37) | Number of patients seen in duration of time (EP) |  |
|  | Time to review patient with checklist compared with time taken to review without checklist (EP) |  |
|  | Evaluate efficiency of infusion preparation (EP) |  |
| Number of tests ordered (EPEFFIC38) | Number of appropriate diagnostic tests ordered per patient of specific condition (EP) | - Patient notes - Patient feedback data |
|  | Number of appropriate tests ordered (EP) |  |
|  | Number of tests ordered (MTDEP) |  |
|  | Patient experience (MTDEP) |  |
| Amount of waste (EPEFFIC39) | Number of medicines returned to pharmacy (EP) | - Records of ‘Drug box’ returns to pharmacy e.g. resus boxes - Inventory orders |
|  | Monetary value of medicines returned to pharmacy (EP) |  |
|  | Drug expenditure (EP) |  |
|  | Time to prepare drugs (EP) |  |
|  | Stock holding (EP) |  |
|  | Horizon scanning (EP) |  |
|  | Budget management (EP) |  |
|  | Needle use / ordering rate per week (MTDEP) |  |
|  | Stock rotation (MTDEP) |  |
|  | Whether systems are in place to identify out-of-date products (MTDEP) |  |
|  | Whether systems are in place to monitor short shelf life drugs (MTDEP) |  |
| Extent of idea waste (EPEFFIC40) | Time spent on policy development (EP) |  |
|  | Review networking activity (EP) |  |
| Amount of patient contact (EPEFFIC41) | Evaluation of patient facing activities (EP) |  |
|  | Review structured portfolio (EP) |  |
|  | Number of patients reviewed (EP) |  |
|  | Number of interventions made (EP) |  |
|  | Time spent with patient (MTDEP) |  |
|  | Time spent with patients when ED busiest (MTDEP) |  |

Table 10. Direct outcomes and outcome indicators: Equitable care

| **Outcomes** | **Outcome indicators: measure…** |  |
| --- | --- | --- |
| Clinical governance and continuity of care (EPEQ20) | Pharmacist input to ED clinical governance meetings and incident investigations (EP) |  |
|  | Pharmacist input to ED incident investigations (EP) |  |
|  | Pharmacist input to development of guidelines and protocols (EP) |  |
|  | Number of policies developed with pharmacist involvement (MTDEP) |  |
|  | Pharmacist input to review of guidelines and protocols (EP) |  |
| Equal treatment based on condition (EPEQ21) | Evaluate for each condition (EP) | - Gender data - Geographical data - Socioeconomic data |
|  | Number of prescriptions given per condition (MTDEP) |  |
|  | Variation in patient treatment based on time of arrival at the ED (MTDEP) |  |
|  | Adherence to local/national guidelines with respect to gender/ geographical/socioeconomic bias (MTDEP) |  |
| Adequacy of treatment prescribed (EPEQ42) | Number of prescribing decisions influenced by patient characteristics (EP) |  |
|  | Adherence to local/national guidelines (EP) |  |
|  | Review whether patient discriminated against with respect to treatment given (EP) |  |
|  | Time taken to give treatment (MTDEP) |  |
|  | Patient journey time (MTDEP) |  |
|  | Re-attendance rates (MTDEP) |  |
|  | Whether treatment is working immediately after being given (MTDEP) |  |
|  | Whether treatment is working 24 hours after being given (MTDEP) |  |
| Appropriateness of treatment (EPEQ43) | Adherence to local/national guidelines (EP) |  |
|  | Adherence to national guidelines (EP) |  |
|  | Review whether patient discriminated against with respect to treatment given (EP) |  |
|  | Patient feedback (MTDEP) |  |
| Harm or effectiveness of medicines prescribed (EPEQ44) | Number of medicines de-prescribed (EP) |  |
|  | Number of medicines acutely de-prescribed (EP) |  |
|  | Number of medicines initiated for a particular clinical cohort (EP) |  |
|  | Rate of adverse drug reactions (MTDEP) |  |
|  | Clinical outcomes in context of medicines prescribed (MTDEP) |  |
|  | Evaluation of disease state (EP) |  |
|  | Number of medicine related admissions (EP) |  |
|  | Whether prescribing according to guidelines so that treatment is funded (EP) |  |
|  | Evaluate pharmacist prescribing activities against local formulary / guidelines for specific conditions (EP) |  |
|  | Evaluate pharmacist prescribing activities against national formulary / guidelines for specific conditions (EP) |  |
| Harm or effectiveness of tests patient subjected to (EPEQ45) | Evaluate appropriateness of tests conducted (EP) | - Patient notes |
|  | Whether tests are clinically relevant (MTDEP) |  |

Table 11. Indirect outcomes: *Evaluated* influence on others

| **Influence on others** | |
| --- | --- |
| **Doctor** | |
| Doctor acknowledgement of pharmacist recommendation (22) |  |
| Doctor acceptance of pharmacist intervention related to (3, 4, 16, 17, 20, 32): | Prevention of medication error (20) |
|  | Optimisation of medication therapy (20) |
|  | Modification of antimicrobial regimen (13) |
|  | Changing medicine prescribed (22) |
|  | Changing dose prescribed (22) |
|  | Changing administration route (23) |
|  | Lack of medicines reconciliation (22) |
|  | Untreated indication (22) |
|  | Incorrect dosage (22) |
|  | Incorrect frequency (22) |
|  | Inappropriate medicine prescribed (22) |
|  | Adverse reactions (13, 22) |
|  | Wrong formulation (22) |
|  | Wrong dose units (22) |
|  | Interactions (22) |
|  | Prescription errors (22) |
|  | Prescriptions without indication (22) |
|  | Laboratory tests (13) |
|  | Medicine prescribed for the wrong patient (3) |
|  | Wrong drug prescribed (3) |
|  | Drug omitted (3) |
|  | Drug / drug class duplication (3) |
|  | Contraindication (3) |
|  | Allergy (3) |
|  | Drug-drug interaction (3) |
|  | Intravenous incompatibility (3) |
|  | Wrong dosage form (3) |
|  | Wrong strength (3) |
|  | Wrong route or technique (3) |
|  | Dose omission (3) |
|  | Extra dose (3) |
|  | Under dose (3) |
|  | Overdose (3) |
|  | Rate too fast or slow (3) |
|  | Wrong frequency (3) |
|  | Wrong duration (3) |
|  | Wrong time (3) |
|  | Wrong therapy scheme (3) |
|  | Inadequate monitoring (3) |
|  | Omission of a regular medication (32) |
|  | Provision of drug information i.e. review of evidence based medicine as it pertains to specific patients and corresponding recommendations made to physicians (10) |
|  | Dosage adjustment (10) |
|  | Suggestion to initiate therapy (10) |
|  | Formulary interchange (10) |
|  | Order clarification (10) |
|  | Change empirical antibiotic for Urinary Tract Infection (11) |
|  | Compatibility issues (10) |
|  | Change route of administration (10) |
|  | Discontinue drug therapy (10) |
|  | Toxicology (i.e. drug identification, recommendations for management of poisonings or overdoses, or suggestions regarding therapeutic drug monitoring) (10) |
|  | Changes in medication due to allergy notification (10) |
|  | Drug therapy duplication prevention (10) |
|  | Drug interaction identification (i.e. prevented or determined by the pharmacist) (10) |
| Medication chart written with pharmacist drug history available (25) | Fewer prescribing errors made per patient (25) |
|  | Fewer prescribing errors made per medication order (25) |
|  | Fewer high-extreme risk prescribing errors made (25) |
|  | Fewer moderate risk prescribing errors made (25) |
|  | Fewer low risk prescribing errors made (25) |
|  | Fewer prescribing errors made related to drug omission (25) |
|  | Fewer prescribing errors made related to Incomplete order (25) |
|  | Fewer prescribing errors made related to Wrong dose (25) |
|  | Fewer prescribing errors made related to Not clinically indicated (25) |
|  | Fewer prescribing errors made related to Not safest or best (25) |
|  | Fewer prescribing errors made related to Wrong drug (25) |
|  | Fewer prescribing errors made related to Contraindication (25) |
|  | Fewer prescribing errors made related to More prescribing errors made related to: |
|  | Fewer prescribing errors made related to Wrong administration time (25) |
|  | Fewer prescribing errors made related to ADR and allergies (25) |
| Selection of antibiotics for Community Acquired Pneumonia | More appropriate selection (33) |
|  | More appropriate antibiotic selection continued when patient admitted (33) |
|  | Greater number of patients administered antibiotics within 4 hours of admission (33) |
|  | Average administration time (door to antibiotic) (33) |
| Selection of antibiotics for uncomplicated Urinary Tract Infections | Increase in percentage of patients prescribed appropriate antibiotic (24) |
|  | Decrease in percentage of patients prescribed inappropriate antibiotic (24) |
| Use of gatifloxacin | Reduction in total number of doses of intra-venous gatifloxacin prescribed (increase in proportionate use) (34) |
| **Nurse** | |
| Nurse acceptance of pharmacist interventions related to: | Rate of drug administration (10) |
|  | Therapeutic drug monitoring that would not result in patient medication order changes |
| Nurse acceptance rate of pharmacist interventions related to: | Incorrect medication preparation (3) |
|  | Incorrect medication administration (3) |
| **Other** | |
| Override of requirement for ED pharmacist to prospectively review prescription (17, 23) |  |
| Clinical decisions made with complete and correct information available | Fewer patients had one or more error in medicines reconciliation when completed by pharmacist (9) |
|  | Errors of incompleteness (9) |
|  | Errors of inaccuracy (9) |
|  | More patients had allergy status was documented when medicines reconciliation was completed by pharmacist (9) |

Table 12. Indirect outcomes: *Presumed* outcome due to influence on others

| **Presumed outcomes** |
| --- |
| Reduced number of medication errors (4, 17)   - Prescribing errors (4) |
| Reduced costs as a result of (4):   - Interventions related to drug-drug or drug-disease interactions, or drug incompatibilities (10) - Therapeutic recommendations (10) - Medication error prevention (10) - Adverse drug event prevention (10) |
| Cost avoidance   - Avoidance of adverse drug event costs (32) |
| Decreased notification time (4) |
| Increased patient compliance due to more affordable medicine being prescribed (20) |
| Delay in availability of patient’s medication (9) |
| Delayed administration of patient’s medication (9) |
| Reduced number of adverse events related to inaccurate or incomplete allergy histories (9) |
| Reduced likelihood of harm for patients admitted to hospital from ED (25) |
| Increased patient safety (17) |
| Reduced risk of harm (17) |
| Identification of preventable adverse drug events (32) |
| Reduction of preventable adverse drug events (32) |
| Reduction in drug harm (32) |

**References**

1. Collignon U, Oborne CA, Kostrzewski A. Pharmacy services to UK emergency departments: a descriptive study. Pharm World Sci. 2010;32:90-6. https://doi.org/10.1007/s11096-009-9347-3.

2. Ernst AA, Weiss SJ, Sullivan At, Sarangarm D, Rankin S, Fees M et al. On-site pharmacists in the ED improve medical errors. Am J Emerg Med. 2012;30:717-25. https://doi.org/10.1016/j.ajem.2011.05.002.

3. Rothschild JM, Churchill W, Erickson A, Munz K, Schuur JD, Salzberg CA et al. Medication errors recovered by emergency department pharmacists. Ann Emerg Med. 2010;55:513-21. https://doi.org/10.1016/j.annemergmed.2009.10.012.

4. Abdelaziz H, Al Anany R, Elmalik A, Saad M, Prabhu K, Al-Tamimi H et al. Impact of clinical pharmacy services in a short stay unit of a hospital emergency department in Qatar. Int J Clin Pharm. 2016;38:776-9. https://doi.org/10.1007/s11096-016-0290-9.

5. Cohen V, Jellinek-Cohen SP, Likourezos A, Lum D, Zimmerman DE, Willner MA et al. Feasibility of a Pharmacy-Based Influenza Immunization Program in an Academic Emergency Department. Ann Pharmacother. 2013;47:1440-7. https://doi.org/10.1177/1060028013502456.

6. Attwood RJ, Garofoli AC, Baudoin MR, Smith VD, Woloszyn AV, Berg AK et al. Impact of emergency department clinical pharmacist response to an automated electronic notification system on timing and appropriateness of antimicrobials in severe sepsis or septic shock in the emergency department. Ann Emerg Med. 2012;1:118. https://doi.org/10.1016/j.annemergmed.2012.06/310

7. Davis LC, Covey RB, Weston JS, Hu BBY, Laine GA. Pharmacist-driven antimicrobial optimization in the emergency department. Am J Health-Syst Pharm. 2016;73:49-56.

8. Fee C, Chen GH. Impact of Pharmacy Review of Emergency Department Medication Orders on Time From Antibiotic Order to Administration for Patients Admitted With Pneumonia. Ann Emerg Med. 2010;56(3):S143-S.

9. Hayes BD, Donovan JL, Smith BS, Hartman CA. Pharmacist-conducted medication reconciliation in an emergency department. Am J of Health-Syst Pharm. 2007;64:1720-3.

10. Lada P, Delgado Jr G. Documentation of pharmacists' interventions in an emergency department and associated cost avoidance. Am J of Health-Syst Pharm. 2007;64:63-8.

11. Lingenfelter E, Drapkin Z, Fritz K, Youngquist S, Madsen T, Fix M. ED pharmacist monitoring of provider antibiotic selection aids appropriate treatment for outpatient UTI. Am J Emerg Med. 2016;34:1600-3. https://doi.org/10.1016/j.ajem.2016.05.076.

12. Okere AN, Renier CM, Tomsche JJ. Evalulation of the Influence of a Pharmacist-led Patient-Centered Medication Therapy Management and Reconciliation Service in Collaboration with Emergency Department Physicians. J Manag Care Spec Pharm. 2015;21:298-306.

13. Randolph TC. Expansion of pharmacists' responsibilities in an emergency department. Am J Healh-syst Pharm. 2009;66:1484-7.

14. Robey-Gavin E, Abuakar L. Impact of Clinical Pharmacists on Initiation of Postintubation Analgesia in the Emergency Department. J Emerg Med. 2016;50(2):308-14. https://doi.org/10.1016/j.jemermed.2015.07.029.

15. Zhang X, Rowan N, Pflugeisen BM, Alajbegovic S. Urine culture guided antibiotic interventions: A pharmacist driven antimicrobial stewardship effort in the ED. Am J Emerg Med. 2017;35:594-8. https://doi.org/10.1016/j.ajem.2016.12.036.

16. Weeks GR, Ciabotti L, Gorman E, Abbott L, Marriott JL, George J. Can a redesign of emergency pharmacist roles improve medication management? A prospective study in three Australian hospitals. Res Social Adm Pharm. 2014;10:679-92. https://doi.org/10.1016/j.sapharm.2013.10.001.

17. Rafie S, Chan T, Castillo E, Humber D, Dunlay R. Evaluation of a pharmacist oversight program for medication use in the emergency department of an academic medical center. Pharmacotherapy. 2011;31:399.

18. Mills PR, McGuffie AC. Formal medicine reconciliation within the emergency department reduces the medication error rates for emergency admissions. Emerg Med J. 2010;27(12):911.

19. Montgomery K, Hall AB, Keriazes G. Pharmacist's Impact on Acute Pain Management During Trauma Resuscitation. J Traum Nurs. 2015;22:87-90.

20. Cesarz JL, Steffenhagen AL, Svenson J, Hamedani AG. Emergency Department Discharge Prescription Interventions by Emergency Medicine Pharmacists. Ann Emerg Med. 2013;61:209-14.

21. Mattson AE, Scherber KJ, Clark SL, Rudis MI. Impact of pharmacist bedside tissue plasminogen activator (TPA) admixture for ischemic stroke in the emergency department. Acad Emerg Med. 2017;24:193-4. https://doi.org/10.1111/acem.13203

22. Mogensen CB, Olsen I, Thisted AR. Pharmacist advice is accepted more for medical than for surgical patients in an emergency department. Dan Med J. 2013;60:6.

23. Chan TC, Castillo EM, Humber DM, Killeen JP. Frequency of Pharmacy Interventions and Emergency Overrides Following Implementation of Electronic Pharmacy Review In the Emergency Department. Ann Emerg Med. 2010;56:112.

24. Kellner FN, Freer C. Impact of a clinical pharmacist on antibiotic selection for treating urinary tract infections in the emergency department. ASHP Midyear Clinical Meeting. 2012;2011:292.

25. deClifford JM, Caplygin FM, Lam SS, Leung BK. Impact of an emergency department pharmacist on prescribing errors in an Australian hospital. Journal of Pharmacy Practice and Research. 2016;46:25-7. doi:http://dx.doi.org/10.1002/jppr.1275.

26. Gosser RA, Arndt RF, Schaafsma K, Dang CH. Pharmacist Impact on Ischemic Stroke Care in the Emergency Department. J Emerg Med. 2016;50(1):187-93. doi:10.1016/j.jemermed.2015.07.040.

27. Mohorn P, Cox H, Grof T. Time to initial antimicrobial therapy in emergency department patients with sepsis improves when a clinical pharmacist participates on the care team. Crit Care Med. 2012;1:129.

28. Perry T, Gama J, Jost P, Tovar J, Olson-Mack L, Rockwell JM. Improving door-to-needle time with direct pharmacy ordering process. Stroke. 2016;47:239

29. Miller K, McGraw MA, Tomsey A, Hegde GG, Shang J, O'Neill JM et al. Pharmacist addition to the post-ED visit review of discharge antimicrobial regimens. Am J Emerg Med. 2014;32:1270-4.

30. Jiron M, Martinez M, Sandoval T, Herrada L. Effect of clinical pharmacist interventions on patient satisfaction in the emergency department: A randomized controlled trial. Pharmacoepidemiol Drug Saf. 2017;26:598. https://doi.org/10.1002/pds.4275.

31. Hatton K, Grice K, Morgan L, Wright D. Evaluation of the Pharmacist Service to the Emergency Department at a Large Teaching Hospital. Pharmacoepidemiol Drug Saf. 2016;25:12-3.

32. Gaskin J, Conyard E. Clinical pharmacist interventions in the emergency department and their impact on preventable adverse drug events and associated cost avoidance. Eur J Hosp Pharm. 2017;24:81. https://doi.org/10.1136/ejhpharm-2017-000640.179.

33. Hanna N, Price CA, Cook B, Graziose G. Pharmacist impact on the appropriate selection and administration time of antibiotics in patients with community acquired pneumonia presenting to the emergency department. ASHP Midyear Clinical Meeting. 2005;40:509.

34. Parris M, Tredway L, Henry VR. Success with pharmacist interventions and quinolone prescribing patterns. ASHP Midyear Clinical Meeting. 2003;3.
