# Supplementary material for: Development of the Manchester framework for the evaluation of emergency department pharmacy services
Source: Int J Clin Pharm. 2022 Apr 21;44(4):930–8. doi: 10.1007/s11096-022-01403-w (PMC9393142; doi:10.1007/s11096-022-01403-w)
Supplement: Supplementary file 2 — Supplementary Material 2 [file 11096_2022_1403_MOESM2_ESM.docx]

## Online Appendix B. Search strategy and search results

**Search strategy**

| Primary terms |  | ‘Emergency department’ | AND | ‘Structure, process and outcome’ | | AND | ‘Pharmacist’ |
| --- | --- | --- | --- | --- | --- | --- | --- |
|  | **OR** |  |  |  |  |  |  |
| Secondary terms |  | Emergency department* |  | Structure* | Interpersonal |  | Pharmacist* |
|  |  | ED |  | Organisation* | Clinical care |  | Pharmacy service |
|  |  | casualty |  | Characteristics | Biomedical care |  | Pharmacy |
|  |  | A&E |  | Resources | Management |  |  |
|  |  | Accident and emergency |  | Staff | Co-ordination |  |  |
|  |  | Emergency room* |  | Equipment | Outcome* |  |  |
|  |  |  |  | Building* | Patient reported outcome* |  |  |
|  |  |  |  | Personnel | Health related outcome* |  |  |
|  |  |  |  | Skills | Health related quality of life |  |  |
|  |  |  |  | Management | Consequence* |  |  |
|  |  |  |  | Skill-mix | Result* |  |  |
|  |  |  |  | Teamworking | Health status |  |  |
|  |  |  |  | Process* | User evaluation |  |  |
|  |  |  |  | Preventative care | Satisfaction |  |  |
|  |  |  |  | Acute illness | Enablement |  |  |
|  |  |  |  | Chronic care | Symptom resolution |  |  |
|  |  |  |  | Interventions | Evaluat* |  |  |
|  |  |  |  | Care delivery | Investigat* |  |  |
|  |  |  |  | Care provision | Impact |  |  |
|  |  |  |  | Interaction | Measure |  |  |
|  |  |  |  | Relationship | Indicator |  |  |
|  |  |  |  | Technical care |  |  |  |

**Literature search results prior to removal of duplicates**

| Database | Search term and limiters used | | | Date of search | Publications identified |
| --- | --- | --- | --- | --- | --- |
|  | ‘Emergency department’ | ‘Structure, process and outcome’ | ‘Pharmacist’ |  |  |
| CINAHL | Abstract | Title | Title | 03/07/2017 | 74 |
| Web of Science Core Collection | Topic | Title | Title | 03/07/2017 | 114 |
| Medline | Abstract | Title | Title | 09/10/2017 | 116 |
| International Pharmaceutical Abstracts | Abstract | Title | Title | 09/10/2017 | 94 |
| Embase | Abstract | Title | Title | 18/12/2017 | 282 |
