# Supplementary material for: Development of the Manchester framework for the evaluation of emergency department pharmacy services
Source: Int J Clin Pharm. 2022 Apr 21;44(4):930–8. doi: 10.1007/s11096-022-01403-w (PMC9393142; doi:10.1007/s11096-022-01403-w)
Supplement: Supplementary file 3 — Supplementary Material 3 [file 11096_2022_1403_MOESM3_ESM.docx]

## Online Appendix C. The Manchester Framework for the evaluation of Emergency Department Pharmacist Services


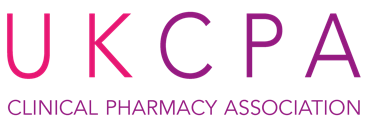

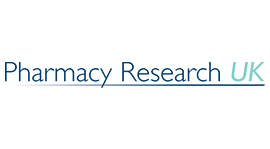

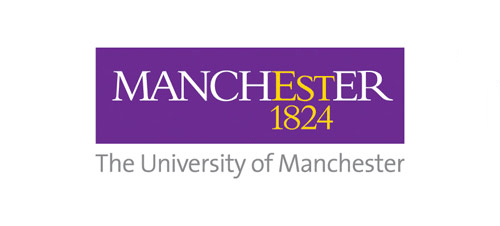


**The Manchester Framework for the evaluation of Emergency Department Pharmacist Services**

**Introduction**

The evaluation of healthcare is important to ensure high-quality care provision and appropriate resource allocation. This is no different for ED pharmacist services, where in some countries pharmacists are providing new types of care to patients with urgent and emergency conditions. The evaluation of both existing and emerging roles is warranted, not least to understand whether and how pharmacists enhance the quality of care provided – or the contrary – but also how those services should develop.

Defined by the Institute of Medicine as six domains, quality care is: safe, effective, patient centred, timely, efficient and equitable. Evaluation of the quality of care involves measurement of outcome indicators, which are the consequence of care. Aside from outcomes, evaluation should also involve consideration of the environment through which care is provided (known as structures), and also actual care delivery (known as processes). Any outcome measurements should be interpreted in the context of both structures and processes, to aid understanding of the origins of those measurements.

This framework comprises three sections. First, Section A presents potential outcome indicators which could – with further development (described later) – be used to measure the quality of ED pharmacist services. The outcome indicators are listed for each of the six quality domains, grouped into different topics of evaluation. In Section B, examples of structures and processes which could be considered when measuring outcome indicators, are listed. Finally, Section C presents an examples of how these different components (structures, processes and outcomes) have previously been used to evaluate the impact of ED pharmacist services. Some examples of data sources which could be used for evaluation studies are also listed.

**Development of the framework**

This framework was developed through a review of relevant literature, and the contribution of ED pharmacists and other healthcare professionals. From five literature databases, 34 studies which had previously evaluated the impact of ED pharmacists were identified. These studies, which originate from different countries including the United States, Australia, Saudi Arabia, Colombia and the United Kingdom, were each reviewed with outcome indicators, structures and processes extracted and collated.

Next, to identify further outcome indicators and example processes, a meeting of seven ED pharmacists was convened at the University of Manchester. The pharmacists also suggested data sources which could potentially be used to evaluate outcome indicators. To gain a different perspective, another meeting was held with other ED healthcare professionals (non-pharmacists) to identify further additional outcome indicators and data sources. Specific participants were: two staff nurses; a nurse practitioner; a physiotherapist; and an occupational therapist.

**Section A. Outcome indicators**

The following six tables list potential outcome indicators for the six domains of quality, grouped into topics of evaluation. As *potential* outcome indicators, they still require further development prior to use in a particular health setting e.g. definition of an acceptable measurement, and reliability and validity testing. Clinicians should recruit researchers with experience in outcome measurement to further develop and measure the indicators presented in this framework.

Some of the outcome indicators might be irrelevant to the role of pharmacists in your country and ED, while others may seem too general. To support global relevance of the framework, indicators have been worded specific enough for their purpose to be understood, but general enough to enable local application. For example, the indicator “Number of new medicines initiated for particular clinical groups” could be used to measure the impact of different types of ED pharmacy service. In those countries where pharmacists have prescribing rights e.g. the United Kingdom, it could be used to evaluate the number of new medicines initiated *by a pharmacist* for particular patients. In other countries where pharmacists do not prescribe, the measure could be used to evaluate the impact of ED pharmacists who review prescriptions written by other healthcare professionals. Another point of note, throughout, the phrase ‘stages of medicines use’ are often described which is used collectively for prescribing, dispensing, administration and monitoring. The collective term was used to collate similar outcome indicators.

| Table 1: SAFE CARE – outcome indicators to evaluate the *safety* of ED pharmacy services i.e. when a pharmacist is part of/contributes to the care team | |
| --- | --- |
| To evaluate… | **You could measure…** |
| The safety/safety impact of medication history taking/medicines reconciliation by a pharmacist | - Whether medicines reconciliation was undertaken - Number of errors in medication history/reconciliation taken by pharmacists e.g. allergy status not documented - How medicine(s) administered in the ED differ from the medicine(s) recorded in the medication history/reconciliation - How medicines reconciliation performed differs from an optimal reconciliation - Delays in medicine(s) use e.g. time taken to gather the correct information from different sources, supply medication, administer medication - Number of adverse events due to errors in medication history/reconciliation taken by pharmacists e.g. if allergy status not documented - How a pharmacist-taken drug history/medicines reconciliation affects doctor prescribing e.g. number of prescribing errors made |
| Pharmacist prescribing | - Number of prescribing errors - Number of adverse events for pharmacist prescriptions - Number of interventions made for pharmacist prescriptions - Re-attendance to ED or General Practitioner and reasons for re-attendance |
| Whether medication supply is timely i.e. to prevent harm | - Time from prescribing to dispensing and administration - Number of missed doses due to delayed supply - Whether or not the requirement for pharmacists to prospectively review prescriptions is overridden |
| The safety of a pharmacist-led vaccination service in the ED e.g. flu | - Number of adverse events |
| Pharmacist response to staff/patient queries which concern safety | - Time from query to response - Satisfaction with response |
| Pharmacists stopping of medicine(s) e.g. to prevent adverse events | - Time until the medicine(s) was re-started - Whether medicine(s) was stopped before arrival at ED - Number of patients for whom medicine(s) was stopped due to an adverse event |
| Medication errors made when a pharmacist is part of the care team | - Number of patients who are subject to an error e.g. prescribing errors or errors which involve a high-risk medicine(s) - Whether pharmacists prevent errors from the reaching the patient - Number of patients who are harmed - If an error occurs, its impact on length of hospital stay |
| Adverse events | - Number and severity of adverse events that are reported - Number of preventable adverse drug events that occur |
| Re-attendance and re-admission | - Number of patients who re-attend the ED ≤72 hours or ≤7 days e.g. with the same or related complaint as their initial visit - Number of patients re-admitted to hospital - How care after discharge differs from the care planned - Number of patients who make an unplanned visit to GP within ≤7 days of attending the ED |
| Adherence by prescribers to local policy and guidelines i.e. to maximise safety | - Prescriber adherence to empirical treatment guidelines - Adherence to local/national antimicrobial guidelines and policies |

| Table 2: EFFECTIVE CARE – indicators to evaluate the *effectiveness* of ED pharmacy services i.e. when a pharmacist is part of/contributes to the care team | |
| --- | --- |
| To evaluate… | **You could measure…** |
| The feasibility of an opportunistic pharmacist-led vaccination service e.g. flu | - Number of patients requesting vaccination - Time required to provide the vaccination service e.g. screen and counsel patients, and administer - Number of vaccinations administered |
| Appropriate use of antimicrobials | - Staff awareness of, and adherence to, local/national antimicrobial guidelines and policies - Number of inappropriate antimicrobial prescriptions e.g. for an unconfirmed UTI - Use of diagnostic tests e.g. procalcitonin and number of antimicrobial prescriptions avoided - Number of viral infections treated with antibiotics - Number of pharmacist interventions for antimicrobial prescriptions - Number of days of inappropriate antimicrobial avoided due to pharmacist intervention - Patient clinical status e.g. whether infections improve and/or re-occur - Re-attendance to ED or GP - Resistance to different antimicrobials for specific infections - Number of patients who die from particular conditions e.g. from sepsis - Number of patients administered antibiotics within 4 hours of admission |
| The appropriate use of Primary Care services rather than inappropriate use of ED | - Number of visits to/contacts with primary care providers per patient - Whether primary care providers believe their service is used appropriately - Number of patients who seek advice from an urgent care telephone service e.g. NHS 111 in the UK - Number of visits to ED per patient over a defined period preceding current visit e.g. 90 days - Number of visits to ED on days 1-30; 31-60; and 61-90 after discharge |
| Effective use of medicine(s) when a pharmacist is part of the care team e.g. prescribes or reviews treatment | *This will depend on the type of medicine being evaluated. For example:*  *To evaluate the use of Tissue Plasminogen Activator, you could measure relevant clinical indicators such as:*   - Whether patient blood pressure is reduced to the required level   *To evaluate the use of analgesia e.g. for trauma or post-intubation, you could measure relevant clinical indicators such as:*   - Number of patients who receive analgesia - Change, and rate of change, in patient pain scores |
| Unnecessary prescribing | - Adherence to national prescribing guidelines/protocols - Whether unnecessary medicine(s) is prescribed - Whether medicine(s) prescribed are supplied/administered - Number of medicines pharmacists advise should be stopped - Number of medicines wasted |
| The cost effectiveness of prescribing | - Drug expenditure e.g. overall and for specific drugs - Number of non-formulary drug requests - Whether drugs prescribed are or are not the cheapest available option |
| The effective use of treatment by patients | - Patients knowledge of their condition and treatment - Number of patients who re-attend the ED with the same complaint as their initial visit, or a related complaint - Number of ED visits/hospital admissions due to poor use of medicines by patients - How patients use their medicine(s) e.g. compliance |
| Whether guidelines, policies and procedures are evidence based | - Pharmacist involvement in guideline/policy development, implementation and audit of their use - The quality of evidence used to develop policies - How local guidelines, policies and procedures compare with national ones - Pharmacist involvement in clinical governance meetings |
| The appropriateness of pharmacist prescribing | - Number of interventions/amendments made for pharmacist prescriptions - Adherence to local/national prescribing guidelines |
| Procedures undertaken by pharmacists | - Number of specific procedures e.g. cannulations, vaccinations, blood samples for therapeutic drug monitoring |
| Mortality and morbidity of patients cared for by teams containing a pharmacist | - Mortality and morbidity rates e.g. for patients of different acuity - Avoidable deaths that are medication related |
| Polypharmacy | - Number of medicines de-prescribed permanently or temporarily - Change in the number of medicines prescribed on arrival to ED compared with discharge - Number of new medicines initiated for particular clinical groups |
| Medication interventions by pharmacists | - Whether doctors/nurses acknowledge pharmacist interventions - Whether doctors accept pharmacists’ medication interventions e.g. suggestions to change a medicine, dose, or time of administration, or prescribe an omitted medicine - Whether nurses accept pharmacists’ medication interventions e.g. to change the administration rate of an intra-venous medicine, or the method used to prepare a medicine |

| Table 3: PATIENT CENTRED CARE – indicators to evaluate the patient centredness of ED pharmacy services i.e. when a pharmacist is part of/contributes to the care team | |
| --- | --- |
| To evaluate… | **You could measure…** |
| Patient satisfaction | - Number of patients dissatisfied with their ED visit after discharge - Patient satisfaction with pharmacist consultation |
| Whether the formulation of prescribed medicine is appropriate | - Whether patients are able to take the prescribed medicine - Patient opinion of formulation administered, including the opinions of particular groups e.g. children or those with a nasogastric tube - Change in patient compliance if a cheaper medicine (e.g. generic) is prescribed - Whether alternative formulations are sourced for patients - Adherence to local/national formulary/guidelines |
| Patient involvement in decision making | - Time taken to education patient on therapy options - Patient contribution to decision making e.g. prescribing decisions - Whether pharmacists involve patients in decision making - Patient satisfaction with decisions made about their care e.g. prescribing decisions |
| Patient education on their condition and treatment | - Patient understanding of their medicines - Patient opinion of being educated on their condition - Patient compliance with therapy after initiation - Number of patient problems with medicines - Medication related re-attendance to ED or General Practitioner (GP) |
| Whether patient respect and dignity is maintained | - Adherence to safeguarding policy - Patient satisfaction with care i.e. whether respect and dignity maintained |

| Table 4: TIMELY CARE – indicators to evaluate the *timeliness* of ED pharmacy services i.e. when a pharmacist is part of/contributes to the care team | |
| --- | --- |
| To evaluate… | **You could measure…** |
| Timeliness of stages in the care pathway | - Time to first assessment for patients with symptoms of severe conditions e.g. shortness of breath in suspected sepsis - For suspected infections, compliance with infection identification tools within a suitable timeframe e.g. Sepsis Six compliance within 1 hour of patient arrival at ED - Time taken for different stages of patient management e.g. from arrival at ED to diagnosis and/or admission to hospital - Time taken to supply and deliver discharge medicines to patients |
| Time to time-critical medicines e.g. antimicrobials, analgesia, Tissue Plasminogen Activator and anti-Parkinsonians | *Relevant clinical indicators*   - Time between arrival at ED or bed (bay) allocation to stages of medicine use e.g. from arrival to antimicrobial prescription or administration - Time between stages of medicine use e.g. time between administration of analgesia by ambulance service and again in the ED; time between antimicrobial prescription and administration - Time from relevant tests or diagnosis to stages of medicine use e.g. from Computer Tomography scan results obtained by clinician to Tissue Plasminogen Activator prescription or administration - Number of delayed or missed doses of medicine e.g. of anti-Parkinsonian medicines - Time taken to change inappropriate discharge medicine to appropriate discharge medicine e.g. for antimicrobials - Patient opinion of time taken to be supplied with time-critical medicine |
| Length of ED and hospital stay | - Length of ED stay e.g. overall and in different areas such as ‘majors’ or ‘resus’ - Length of hospital stay both overall and for different inpatient departments - Length of hospital stay for specific clinical groups e.g. those who experience medicines related admissions |
| Hospital admissions | - Number of admissions - Number of medication related admissions - Number of attendances to ambulatory care clinics - Number of patients signposted to other services who re-attend the ED - The quality of pharmacist referrals to specialists - Whether admissions are avoided |
| Timeliness of a pharmacist-led vaccination service e.g. flu | - Time taken to screen patients for eligibility - Time taken to administer influenza vaccination - Number of patients vaccinated - Time from offering patients the vaccination to administration - Number of staff vaccinated |

| Table 5: EFFICIENT CARE – indicators to evaluate the *efficiency* of ED pharmacy services i.e. when a pharmacist is part of/contributes to the care team | |
| --- | --- |
| To evaluate… | **You could measure…** |
| Use of ‘Patient’s Own Drugs’ (PODs) to reduce use of hospital medicines | - Number of patients who have/use PODs in the ED/inpatient wards if admitted to hospital - Expenditure on PODs (i.e. regular medicines) compared with acute medication (i.e. newly prescribed in ED) - Whether patient’s prescriptions in primary care are regular (i.e. routine supply by community pharmacy) |
| Stock availability | - Time from prescription to administration of items stocked/not stocked on the ward - Number of medicine requests made by ED to pharmacy - Number/type of medicines borrowed from other (non-ED) departments/wards - Whether medicines not immediately available are ordered - Medicine stock levels in the ED - Utilisation of nursing personnel to find required drugs in the ED |
| The time efficiency of care | - Number of patients seen in a particular timeframe - Time to review patients - Efficiency of medicines preparation e.g. intravenous infusions |
| Amount of waste | - Number/value of medicines returned to hospital pharmacy - Number of needles used by pharmacists compared with those usually ordered per week - Number of appropriate/inappropriate tests ordered - Whether time spent on development of policies which limit waste is sufficient |
| Amount of patient contact with pharmacist | - Number of patients reviewed - Number of interventions made - Time spent with patients |
| Impact of interventions on expenditure | - Cost impact of interventions e.g. those which prevent medication error or optimise therapy |

| Table 6: EQUITABLE – indicators to evaluate the *equitability* of ED pharmacy services i.e. when a pharmacist is part of the care team | |
| --- | --- |
| To evaluate… | **You could measure…** |
| The equitability of clinical governance | - Pharmacist contribution to ED clinical governance meetings and investigations e.g. of incidents - Pharmacist contribution to development and review of guidelines and protocols e.g. how many they helped to develop |
| The equitability of care provided to different patient groups | *For different patient groups e.g. those with different diagnoses or protected characteristics*   - Whether tests undertaken are appropriate e.g. clinically relevant - Whether medicines use differs e.g. treatment prescribed as per local/national guidelines, number of medicines de-prescribed (temporarily/permanently), time taken to administer or supply treatment, how quickly treatment is effective - Whether prescribed treatment is funded e.g. for some diagnoses but not others - Whether treatment varies according to time of arrival at the ED - The safety of medicines use e.g. number of adverse drug reactions, medicines related hospital admissions - Length of stay e.g. time from arrival at ED to discharge from ED/hospital - ED re-attendance rates |

**Section B. Structures and processes**

Structures are the environment through which care is delivered, with processes being actual care provision. As described in the introduction, both should be recorded so that outcome measurements can be interpreted in context e.g. what might be responsible for any observed change. As described further in Section C, both are often reported in published evaluation studies, to help the reader understand the context.

As well as their role in interpreting outcome measurements, structures and processes can also be used to identify relevant outcome indicators. For example, if ED pharmacists do not prescribe (a process), then outcome indicators of pharmacist prescribing would be irrelevant. Hundreds of structures and processes were identified through development of the framework, with some example structures given in Table 7 and processes in Table 8.

| Table 7: Structures of the ED* – for the evaluation of ED pharmacist impact you could consider… | |
| --- | --- |
| Category | **Example** |
| Type of department and areas within | Type 1 ED (UK NHS nomenclature for a major ED) with resuscitation facilities |
| Size of department | Number of visits |
| Specialisms of department | Trauma centre |
| Facilities | Care pathways |
| Pharmacy facilities | Pre-packed medicines |
| Recommended resources | Medicines formulary |
| Patient population | Acuity e.g. number of patients initially seen in resuscitation |
| Pharmacy systems | Systems to triage patients to a pharmacist |
| Pharmacist service | Location from where service provided e.g. ED or central hospital pharmacy |
| Interaction with other ED staff | Collaboration with doctors |
| Interaction with patient’s representative/carer | Offered information to support medication history taking |

*Some structures of the wider care organisation were also identified and so should also be considered e.g. the type of hospitals

| Table 8: Patient specific processes* – for the evaluation of ED pharmacist impact you could consider… | |
| --- | --- |
| Category | **Examples: the pharmacist…** |
| History taking | - Took drug histories - Took a full medical history |
| Clinical examinations | - Performed clinical examinations - Reviewed the findings of clinical examinations |
| Investigations, tests and procedures | - Reviewed the results of urine cultures - Reviewed the results of pregnancy tests |
| Diagnosis | - Diagnosed patients - Educated and trained patients about their diagnosis |
| Management planning | - Involved patients in the development of a plan - Made a plan which was respectful of patient beliefs and decisions |
| Treatment | - Prescribed medicines - Checked the clinical appropriateness of prescriptions |
| Monitoring | - Monitored vital signs - Ordered vancomycin serum levels |
| Discharge/admission to hospital | - Prescribed discharge medicines - For admitted patients, wrote an inpatient drug chart |

*General processes were also identified and so should also be considered e.g. guideline development

**Section C. Methods for evaluation**

An example of how the impact of ED pharmacists can be evaluated is described below, using an example study which measured outcome indicators while also considering structures and processes.

*“Formal medicines reconciliation within the emergency department reduces the medication error rates for emergency admissions.” (Mills and colleagues, 2010)*

In this study, researchers measured the impact of a pharmacist intervention on 24-hour medicine reconciliation rate (*outcome indicator 1),* prescription chart initiation (*outcome indicator 2)* and prescribing error rates (*outcome indicator 3)*. The specific intervention had an ED pharmacist prescriber complete medicine reconciliation (*process*), and where appropriate write an inpatient drug chart (*process*), for 50 ED patients prior to their transfer (e.g. to an inpatient ward). For comparison, data for standard care (i.e. care prior to the intervention) was also collected for 50 patients. In the study, the environment of care provision is also described e.g. how the hospital is a major secondary care facility with 24,000 ED visits per annum (*structure)* and the patient characteristics of both baseline and intervention groups (*structure)* which were compared and no differences identified.

For outcome indicator 1, medicine reconciliation completed within 24-hours of admission increased from 50% for standard care (n=25) to 100% for the intervention cohort (n=50). For outcome indicator 2, prescription chart initiation in the ED increased from 6% (n=3) to 80% (n=40); and for outcome indicator 3, prescribing error rate was reduced from 3.3 errors to 0.04 errors per patient.

The above measurements can be interpreted in the context of structures and processes. For example, the change in outcome indicator measurements between the groups was thought due to the pharmacist intervention e.g. provide medicine reconciliation (process). As described in Section B, that change in process may have informed selection of outcome indicators i.e. those which would be sensitive to the intervention. Through capture of the environment where care was provided (structures) e.g. patient characteristics, Mills and colleagues were able to evaluate whether any difference in outcome indicator measurement was due to differences in the patient cohorts. They concluded there were no statistical differences between the cohorts, supporting the conclusion that the difference in outcome indicator measurements was due to pharmacist processes.

In the study by Mills’ and colleagues, data was collected from prescription charts. Other data sources of data for outcome indicator measurement include:

- Medical notes
- Medication administration records
- Pain and function score charts
- Trauma scoring systems
- Hospital incident reports
- ‘Near-miss’ error logs
- Hospital datasets which concern mortality and morbidity
- Socioeconomic records
- Staff
- Patients
- General Practice (i.e. primary care) records
